# Differential Therapeutic Effect of Extracellular Vesicles Derived by Bone Marrow and Adipose Mesenchymal Stem Cells on Wound Healing of Diabetic Ulcers and Correlation to Their Cargoes

**DOI:** 10.3390/ijms22083851

**Published:** 2021-04-08

**Authors:** Margherita Pomatto, Chiara Gai, Federica Negro, Massimo Cedrino, Cristina Grange, Elena Ceccotti, Gabriele Togliatto, Federica Collino, Marta Tapparo, Federico Figliolini, Tatiana Lopatina, Maria Felice Brizzi, Giovanni Camussi

**Affiliations:** 1 Department of Medical Sciences, University of Turin, 10126 Turin, Italy; margheritaalbacarlotta.pomatto@unito.it (M.P.); chiara.gai@unito.it (C.G.); cristina.grange@unito.it (C.G.); elena.ceccotti@unito.it (E.C.); gabriele.togliatto@unito.it (G.T.); marta.tapparo@unito.it (M.T.); t.lopatina@gmail.com (T.L.); 2 Unicyte Srl, 10126 Turin, Italy; federica.negro@fmc-ag.com; 3 2i3T Scarl University of Turin, 10126 Turin, Italy; massimo.cedrino@unito.it (M.C.); federico.figliolini@unito.it (F.F.); 4 Laboratory of Translational Research in Paediatric Nephro-urology, Fondazione Ca’ Granada IRCCS Ospedale Maggiore Policlinico, 20122 Milan, Italy; federica.collino@gmail.com

**Keywords:** wound healing, MSC, exosome, extracellular vesicle, diabetes, adipose, bone marrow, mesenchymal, therapy, cargo

## Abstract

Extracellular vesicles (EVs) derived from mesenchymal stem cells isolated from both bone marrow (BMSCs) and adipose tissue (ADSCs) show potential therapeutic effects. These vesicles often show a similar beneficial effect on tissue regeneration, but in some contexts, they exert different biological properties. To date, a comparison of their molecular cargo that could explain the different biological effect is not available. Here, we demonstrated that ADSC-EVs, and not BMSC-EVs, promote wound healing on a murine model of diabetic wounds. Besides a general similarity, the bioinformatic analysis of their protein and miRNA cargo highlighted important differences between these two types of EVs. Molecules present exclusively in ADSC-EVs were highly correlated to angiogenesis, whereas those expressed in BMSC-EVs were preferentially involved in cellular proliferation. Finally, in vitro analysis confirmed that both ADSC and BMSC-EVs exploited beneficial effect on cells involved in skin wound healing such as fibroblasts, keratinocytes and endothelial cells, but through different cellular processes. Consistent with the bioinformatic analyses, BMSC-EVs were shown to mainly promote proliferation, whereas ADSC-EVs demonstrated a major effect on angiogenesis. Taken together, these results provide deeper comparative information on the cargo of ADSC-EVs and BMSC-EVs and the impact on regenerative processes essential for diabetic wound healing.

## 1. Introduction

Extracellular vesicles (EVs) are a part of the cell secretome which mediates cell-to-cell communication and cell biological effects. EVs are membrane vesicles secreted by almost all living cells and their classification and nomenclature were set off by the International Society of Extracellular Vesicles (ISEV) [1,2]. EVs exert their biological functions by carrying bioactive molecules to target cells and their cargo includes proteins, nucleic acids and lipids [3]. EVs derived from stem cells have been widely studied for their pro-regenerative properties. Mesenchymal stem cell (MSC)-EVs in particular have been isolated by numerous sources and their regenerative properties have been deeply investigated [4,5]. Among different origins, MSCs isolated from bone marrow (BMSCs) are the most commonly used stem cells in clinical trials [5]. On the other hand, MSCs isolated from adipose tissue (ADSCs) have several advantages in terms of their isolation process, including the abundancy of their tissue of origin, and the easier and less invasive drawing procedure [6]. Both BMSC and ADSC-EVs have been extensively studied, showing potential therapeutic effects in a variety of preclinical experimental models [4]. In fact, both types of EVs share common biological activities and their direct comparison demonstrated similar beneficial effects on accelerating physiological cutaneous wound healing [7]. Wound healing is a complex and dynamic process contributing to a well-orchestrated cutaneous injury repair, and involving many different types of cells, such as endothelial cells, fibroblasts, and keratinocytes [8]. MSC-EVs can orchestrate all phases of skin wound healing due to their ability to modulate several processes involved in migration, inflammation and proliferation of various cells including fibroblasts, immune cells and keratinocytes, and by ameliorating scarring [9]. In fact, recent evidence demonstrates that ADSC and MSC-EVs can improve the cutaneous wound healing by promoting fibroblast and keratinocyte migration and proliferation, collagen deposition, neovascularization, and macrophage polarization into M2 phenotype [10,11,12,13,14]. In a clinical setting, the therapeutic efficacy on wound healing is essential for treating chronic wounds, where the physiologic healing process is impaired. Diabetes mellitus is a primary cause of impaired angiogenesis leading to reduced wound healing. Diabetic wounds represent a worldwide medical problem and a substantial social and economic burden. In the diabetic setting, pathophysiological mechanisms involving cellular dysfunction, inflammation, hypoxia, neuropathy, impaired angiogenesis, and neovascularization impair the healing process [9,15]. Several studies demonstrate that ADSC-EVs can accelerate healing of diabetic skin wounds [16,17], whereas numerous efforts have been made to increase the therapeutic activity of BMSC-EVs, including cell culture preconditioning with deferoxamine [18], melatonin [19], atorvastatin [20], or overexpression of lncRNA H19 [21]. These data highlight the imbalanced therapeutic effect of BMSC and ADSC-EVs on diabetic skin wound healing suggesting different properties and clinical potential associated with these two types of MSC-EVs. Previous studies have shown that the secretome of MSCs derived from different tissues, such as BMSCs and ADSCs, is significantly different [22,23,24]. Moreover, it has been reported that ADSC and BMSC-EVs play different biological effects in different clinical contexts, such as cancer [25], cardiac [26], and Alzheimer’s disease [27]. Thus, it is unclear if the effect of BMSC and ADSC-EVs on skin wound healing in presence of chronic diabetic condition could be similar. To date, no reports have directly compared the therapeutic efficacy of BMSC and ADSC-EVs on diabetic skin wounds. For this reason, we treated skin wounds in a mouse diabetic model with the same dose of EVs isolated by BMSCs and ADSCs in order to evaluate their therapeutic potential. The results demonstrated that ADSC-EVs, but not BMSC-EVs, accelerated wound closure. Since the biological activity of EVs is due to the transfer of their cargo to target cells, we analyzed and compared the cargo of EVs isolated from different sources to highlight differences potentially relevant for their therapeutic application. Baglio et al. [28] performed a deep sequencing of the small RNA profile of EVs derived from BMSCs and ADSCs demonstrating how BMSC and ADSC-EVs have a similar RNA composition but differ in their tRNA content. Here, we performed a bioinformatic analysis of protein and miRNA content of MSC-EVs in order to compare the cargo and verify their target pathway similarities. The results demonstrated that EVs shared the majority of their cargo but with relevant differences in selective enrichment. In fact, molecules carried only by ADSC-EVs were mainly associated to angiogenesis whereas BMSC-EV content was mainly linked to proliferative processes. Finally, we performed in vitro analyses to evaluate the biological effect of EVs on different cell types involved in wound healing processes. Our results confirmed their different biological effect on proliferation, viability and migration of fibroblasts, keratinocytes and endothelial cells. Importantly, BMSC-EVs showed a prevalent effect on cell proliferation and viability, while ADSC-EVs showed a significant improved ability to promote endothelial cell migration and angiogenesis. 

To our knowledge, this is the first work that directly compares the therapeutic activity of BMSC and ADSC-EVs on wound healing in a murine model of diabetes and that correlates their different molecular content to explain their distinct biological effect. The identification of a more appropriate EV source for accelerating diabetic wound healing and the recognition of the mechanisms of action could be of particular relevance to develop adequate therapeutic strategies in humans.

## 2. Results

### 2.1. Experimental Results

#### 2.1.1. Characterization of BMSC and ADSC-EVs

**Figure 1:**
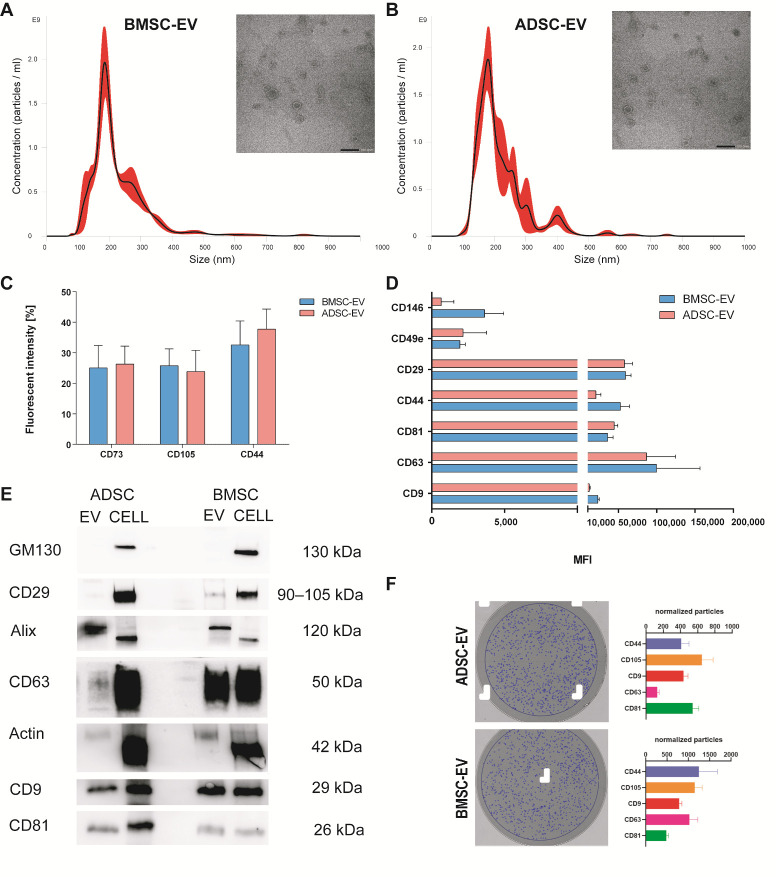
BMSC and ADSC-EV Characterization. EVs were isolated by BMSCs and ADSCs and analyzed using different techniques. Representative Nanoparticle tracking analyses showing the size distribution and representative Transmission electron microscopy of EVs derived from BMSCs (**A**) and ADSCs (**B**) with scale bar 100 nm; (**C**) flow cytometry analysis (FACS) of EVs for surface proteins CD73, CD105 and CD44 showing the percentage of fluorescent intensity; (**D**) MACS multiplex bead-based flow cytometry assay of different surface markers, only expressed markers are shown as mean fluorescent intensity (MFI); (**E**) representative Western blot analysis of integrin β1 (CD29), Actin, exosomal markers CD63, CD9, CD81, Alix, and intracellular marker GM130 as negative control for exosomes in BMSC and ADSC-EVs and cells of origin; (**F**) Interferometry images of a representative anti-CD9 capture spot post-scan for ADSC-EVs (upper-left) and BMSC-EVs (lower-left). Blue circles indicate EVs detected by interferometry. Histograms show the number of normalized particles counted by interferometry for each capturing antibody (CD9, CD63, CD81, CD44, CD105).

EVs were isolated by BMSCs and ADSCs and characterized according to the criteria suggested by the ISEV position paper [1] (Figure 1). The NanoSight analysis demonstrated that BMSC and ADSC-EVs have a similar size distribution profile, with a mean size of 207 ± 36 nm and 211 ± 24 nm, respectively (Figure 1A,B). The concentration measurement revealed that both cell types have a similar EV isolation yield corresponding to 3.44 ± 1.92 × 10^4^ EVs/cell for BMSCs and 3.17 ± 0.74 × 10^4^ EVs/cell for ADSCs. Transmission electron microscopy (TEM) analysis showed a similar round morphology typical of EVs for both samples (Figure 1A,B). 

EVs were characterized using flow cytometry assay (FACS) for the expression of surface proteins (Figure 1C,D). Both EVs expressed MSC markers CD73, CD105 and CD44 (Figure 1C). Moreover, the analysis using MACS multiplex bead-based flow cytometry assay showed that both types of EVs expressed typical MSC markers CD146, CD49e and CD29, exosomal positive tetraspanins (CD81, CD63, CD9) and confirmed the presence of CD44 (Figure 1D). The mesenchymal origin was further confirmed by the absence of expression of the endothelial marker CD31 and the epithelial marker CD326 (data not shown). The measurement of their total protein content revealed a similar cargo corresponding to 26.3 ± 8.1 µg/10^9^ EVs and 21.9 ± 12.6 µg/10^9^ EVs for BMSC-EVs and ADSC-EVs respectively (Figure S2). The expression of typical exosomal markers was verified using western blot (Figure 1E, Supplementary Figure S1). Both EVs expressed exosomal surface markers CD63, CD81, CD9, and typical proteins as Alix, integrin β1 (CD29), and actin, but they failed to express the intracellular GM130 protein which is characteristic of their cells of origin.

Furthermore, the recently developed Exoview technology was used to characterize ADSC and BMSC-EVs confirming the presence of several EV markers on both types of EVs. For that, chips having three capturing spots with antibodies anti-CD9, anti-CD63, anti-CD81, anti-CD44, anti-CD105, and anti-mouse IgG as negative control were used. After an overnight incubation with EVs, chips were scanned and images of captured EVs were taken and used for image interferometry measurement. The number of EVs in the range of 50–200 nm captured for each spot were counted and normalized for the number of events counted on the same pre-scanned chip in order to eliminate background signal. As shown in Figure 1F, both EV types were positive for exosomal markers (CD63, CD9, CD81) and MSC markers (CD44, CD105). Besides confirming protein expression from FACS and Western Blot analysis, ExoView technology allowed the detection of marker co-expression on EV by incubating the chips with fluorescent antibodies for CD9, CD63, and CD81. As shown in Supplementary Figure S3, EVs captured on all chips are also stained by CD9, CD63, and CD81 fluorescent antibodies, confirming that several markers are colocalized on the same EV particle.

Taken together, the characterization performed using several techniques demonstrated that BMSC and ADSC-EVs share several features, including morphology, shape, size, exosome and MSC protein.

#### 2.1.2. BMSC and ADSC-EVs Exerted Different Therapeutic Action on Wound Healing in a Mouse Model of Diabetic Ulcers

**Figure 2:**
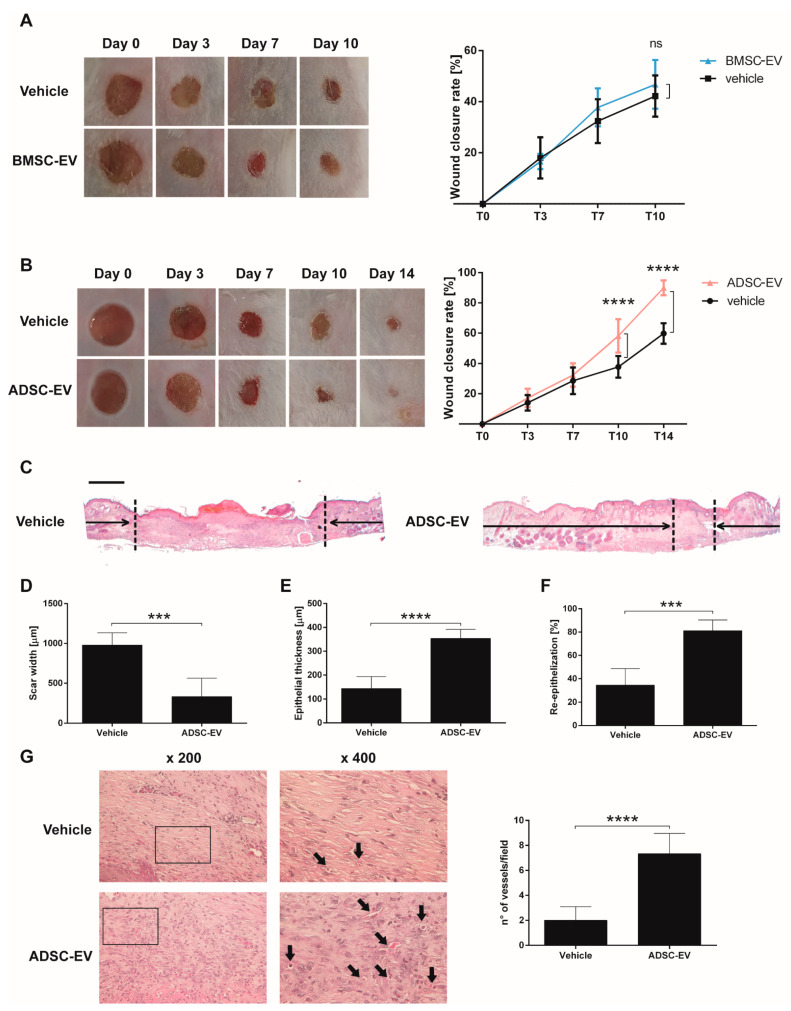
Therapeutic Effect of BMSC and ADSC-EVs on a Mouse Model of Diabetic Ulcers. (**A**) Representative photographs of full-thickness excisional wound treated with carboxymethylcellulose (vehicle) or carboxymethylcellulose and BMSC-EVs (BMSC-EV) and quantification of wound closure rate at 0, 3, 7 and 10 days expressed as percentage of the original wound size at day 0. ns: not statistically significant difference between treatment with vehicle or BMSC-EV; (**B**) Representative image of wounds treated with carboxymethylcellulose (vehicle) or carboxymethylcellulose and ADSC-EVs (ADSC-EV) and quantification of wound closure rate at 0, 3, 7, 10 and 14 days expressed as percentage of the original wound size at day 0. ****: *p* < 0.0001 between treatment with vehicle or ADSC-EV; (**C**) Transmitted light representative images of H&E-stained sections of wounded skin sections at 14 days treated with carboxymethylcellulose (vehicle) or carboxymethylcellulose and ADSC-EVs (ADSC-EV). The black arrows point out the scar edges. Scale bar 200 μm; Quantitative analysis of the scar width (**D**), epithelial thickness (**E**) (µm) and percentage of re-epithelization (**F**) induced by ADSC-EVs treatment in comparison to treatment with vehicle alone at 14 days. *** *p* < 0.005, **** *p* < 0.0001 versus vehicle; (**G**) Representative images of H&E staining at different magnification and quantification of the number of vessels present in wound sections. Black arrows indicate vessels in the micrograph. ****: *p* < 0.0001 between treatment with vehicle or ADSC-EV.

In order to evaluate the therapeutic activity of MSC-EVs in the diabetic wound healing process, an in vivo model of diabetic ulcers was used. Consistent with previous observations [29], the diabetic model showed a slower wound healing process compared to non-diabetic mice (Figure S4). To evaluate the therapeutic potential, EVs were dispersed in a carboxymethylcellulose dressing, used as a vehicle, and applied on the wound. The dressing allows a prolonged release of EVs into the wound over time. The EV dose per treatment corresponded to 1 × 10^9^ EVs repeated every three days. At day 10, our end-point, we noticed that BMSC-EVs were not effective and did not reduce the wound closure rate in comparison to the vehicle alone. Therefore, the experiment was stopped (Figure 2A). On the contrary, ADSC-EVs reduced the size of the wound and the observational period was prolonged till day 14 (Figure 2B). At this timepoint, ADSC-EV treatment almost completely closed the wounds and the effect was statistically significant in comparison with the vehicle treatment. Then, animals were sacrificed and wound sections were H&E stained. The histological analysis confirmed the therapeutic effect of ADSC-EVs in comparison with vehicle. ADSC-EVs reduced the scar width (Figure 2C,D) and increased the epithelial thickness (Figure 2E) and the percentage of re-epithelization (Figure 2F). Angiogenesis is essential for the wound healing process and the histological analysis demonstrated that the treatment with ADSC-EVs increased the number of vessels compared to vehicle alone (Figure 2G and Figure S5).

Taken together, the evaluation of BMSC and ADSC-EV activity on an in vivo model of diabetic ulcer revealed that only ADSC-EVs, but not BMSC-EVs, can accelerate wound closure. In fact, ADSC-EVs reduced the wound and the size of the scar and increased epithelial thickness, re-epithelization and the number of vessels compared to vehicle.

#### 2.1.3. BMSC and ADSC-EV Cargo Analysis Revealed Different Target Pathways 

The different biological effect exerted by ADSC and BMSC-EVs on diabetic ulcers in vivo led us to analyze and compare their miRNA and protein content. Our research group already published several data on single MSC-EV cargo analysis [30,31,32]. Here, these pre-existing datasets were compared. To this purpose, a bioinformatic analysis was performed to investigate and possibly correlate EV content and the biological effect on diabetic skin wound healing.

**Figure 3:**
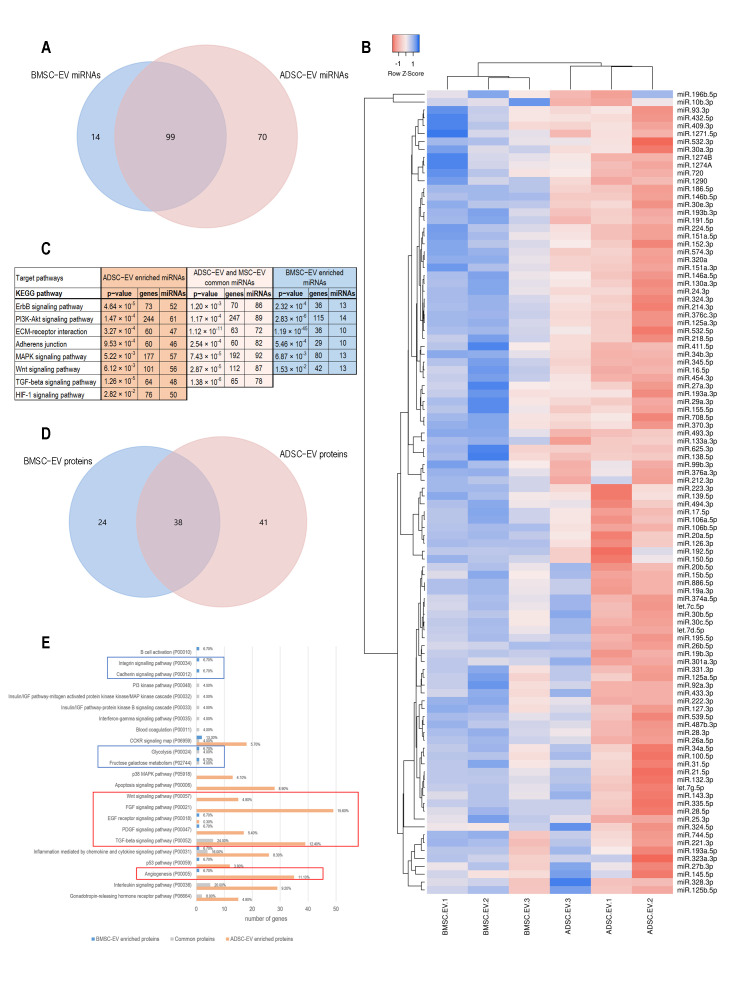
Molecular Analysis of ADSC and BMSC-EV Content. (**A**) The Venn diagram compares the lists of miRNAs carried by ADSC and BMSC-EVs; (**B**) The heatmap shows the clustering of the groups of miRNAs carried by both ADSC and BMSC-EVs. Row Z score of −1 (red) correlates to low Ct values and Z score of +1 (blue) correlates to high Ct values; (**C**) The table shows the most relevant results of target pathway analysis for the lists of miRNAs exclusively carried by ADSC-EVs, miRNAs carried by either ADSC and BMSC-EVs, and miRNAs exclusively carried by BMSC-EVs. The first column shows KEGG pathways, the other columns report p-value, the number of target genes in the pathway, and the number of miRNAs; (**D**) The Venn diagram compares the lists of proteins carried by ADSC and BMSC-EVs; (**E**) The graph shows the target pathways for the proteins only carried by BMSC-EVs (blue bars), carried by both ADSC and BMSC-EVs (grey bars), and only carried by ADSC-EVs. X axis shows the number of genes involved in each pathway, labels on the right show the percent of gene hits against total number of pathways. Panther pathway code is shown next to the pathway name. Signaling pathways related to ADSC-EV proteins are shown in orange, pathways related to BMSC-EV proteins are shown in blue, pathways related to common proteins are shown in grey. Red and blue boxes highlight pathways mainly associated to ADSC-EVs or BMSC-EVs, respectively.

Although miRNAs carried by EVs represent a small fraction of the RNA cargo of EVs, it has been shown that they play a key role in modulating target cell functions [28]. The comparison of miRNAs carried by ADSC and BMSC-EVs showed that 99 miRNAs were present in both types of EVs, while 14 miRNAs were only detected in BMSC-EVs and 70 miRNAs only expressed in ADSC-EVs (Figure 3A and Table 1 and Supplementary Table S1). To better investigate the differences between the two EV types, a clustering analysis on the 99 common miRNAs was performed. As shown in Figure 3B, no significant differences in the expression pattern of ADSC and BMSC-EV miRNAs were detected, with the exception of a general lower content in BMSC-EVs in comparison to ADSC-EVs suggested by the low Row Z-score. The bioinformatic analysis of pathways significantly correlated with miRNAs expressed by both MSC-EVs or exclusively by BMSC or ADSC-EVs was then performed. To this end, miRpath tool [33] was used for identifying numerous KEGG pathways statistically correlated with the miRNAs identified in the three groups (Supplementary Tables S2–S4). The KEGG pathways more relevant for wound healing, such as tissue regeneration, cell proliferation, and angiogenesis, were selected and are shown in Figure 3C. We noticed that several relevant signaling pathways were targeted by the commonly expressed miRNAs in the three groups: the EGFR receptor (ERBB2) signaling pathway and its downstream PI3K/Akt signaling cascade, which mediates endothelial cells migration and proliferation leading to angiogenesis; the ECM-receptor interaction and adherents junction pathways, which are essential for cell adhesion and motility; the MAPK signaling pathway which regulates cell survival, differentiation, and proliferation; and the Wnt signaling pathways, which is involved in cell proliferation and angiogenesis. Interestingly, TGF-β signaling pathway was only targeted by ADSC-EVs and commonly expressed miRNAs, whereas HIF-1α signaling pathway was exclusively targeted by ADSC-EV specific miRNAs.

Protein analysis of ADSC and BMSC-EVs is shown in the Venn diagram reported in Figure 3D demonstrating that 38 proteins were carried by both types of EVs, while 24 proteins were only carried by BMSC-EVs and 41 proteins were only present in ADSC-EVs (Table 2). The evaluation of target pathways correlated with proteins carried by MSC-EVs was performed using Panther pathway tool. Pathways identified as significantly correlated to each group are shown in Figure 3E. ADSC-EV proteins showed a higher interconnection compared to the other groups with several pathways involved in angiogenesis (Wnt, FGF, EGF receptor, PDGF, TGFβ, and angiogenesis). Interestingly, both ADSC-EV proteins and miRNAs targeted Wnt and TGFβ pathways. On the contrary, proteins enriched only in BMSC-EVs were correlated to cell adhesion (integrin and cadherin) and metabolic processes (glycolysis and fructose galactose metabolism).

Taken together, these results demonstrated that BMSC and ADSC-EVs shared the majority of their cargo and that molecules specifically expressed by BMSC-EVs and ADSC-EVs correlate to their specific biological action. These differences in the miRNA and protein content can support their different therapeutic effect observed in the in vivo experiments.

#### 2.1.4. A Direct Comparison of BMSC and ADSC-EVs Revealed Different Beneficial Effects In Vitro

**Figure 4:**
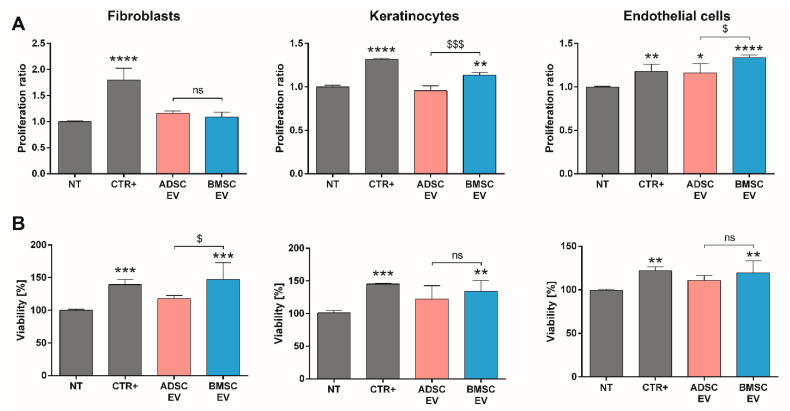
In Vitro Analysis of MSC-EVs on Proliferation and Viability of Cells Involved in Wound Healing Process. (**A**) Proliferation assay of different cell types (fibroblasts, keratinocytes, and endothelial cells) after 24 h of treatment detected using BrdU proliferation assay and expressed as ratio with respect to untreated cells (NT); (**B**) viability assessment on different cell types (fibroblasts, keratinocytes, and endothelial cells) after 48 h of treatment detected using AlamarBlue reagent and expressed as percentage with respect to untreated cells (NT). Cells were treated with following stimuli: DMEM 0% FBS as negative control for untreated cells (NT), DMEM 0% FBS with 50,000 EVs/cell for BMSC or ADSC-EVs (BMSC or ADSC-EV) and different positive controls (CTR+) for each cell type (MCDB131 medium for endothelial cells, FGM-2 Growth Media for fibroblasts and KGMTM Gold Keratinocyte Growth Medium for keratinocytes). Statistical analysis was performed comparing each sample with NT (*: *p* < 0.05, **: *p* < 0.01, ***: *p* < 0.005, ****: *p* < 0.001) or comparing BMSC-EV and ADSC EV ($: *p* < 0.05, $$$: *p* < 0.005, ns: not statistically significant).

To confirm the different activities suggested by bioinformatic analysis, the effects of BMSC and ADSC-EVs were evaluated on the most relevant cell types involved in skin wound healing, including fibroblasts, keratinocytes, and endothelial cells. We demonstrated that both EVs failed to promote the proliferation of fibroblasts. Conversely, BMSC-EVs induced keratinocyte proliferation, whereas although both BMSC and ADSC-EVs promoted endothelial cell proliferation, BMSC-EVs were most effective (Figure 4A). Looking at the ability of EVs to support cell viability, we demonstrated that BMSC-EVs, but not ADSC-EVs, were able to promote fibroblast, keratinocyte, and endothelial cell viability (Figure 4B). These data showed that BMSC-EVs can improve fibroblast metabolic activity and stimulate viability, but the effect was not sufficient to induce proliferation. 

**Figure 5:**
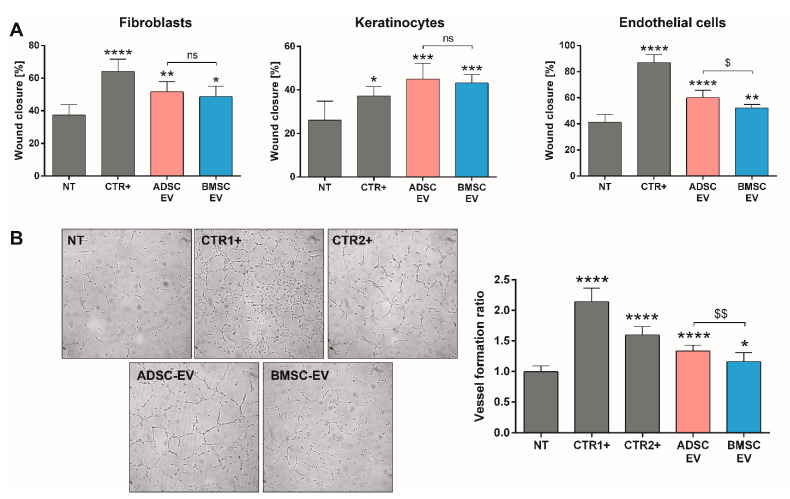
In Vitro Analysis of MSC-EVs Activity on Migration of Cells Involved in Wound Healing Process and Vessel Formation. (**A**) Scratch test assay on different cell types (fibroblasts, keratinocytes and endothelial cells) for measuring the pro-migration effect of MSC-EVs. The wound closure was measured 24 h after treatment and expressed as percentage of the initial wound area; (**B**) representative micrographs and quantitative analysis of capillary-like structure formation test on endothelial cells treated for 24 h. Capillary-like structures length was measured and expressed as ratio in comparison to untreated cells (NT). Cells were treated with DMEM 0% FBS for untreated cells (NT), DMEM 0% FBS with 50,000 EVs/cell for BMSC or ADSC-EVs (BMSC or ADSC-EV) and different positive controls (CTR+) for each cell type (MCDB131 medium for endothelial cells, FGM-2 Growth Media for fibroblasts, and KGMTM Gold Keratinocyte Growth Medium for keratinocytes). For capillary-like structures formation test, MCDB131 medium was used as CTR1+ and 0.1 µg/mL EGF as CTR2+. Statistical analysis was performed comparing each sample with NT (*: *p* < 0.05, **: *p* < 0.01, ***: *p* < 0.005, ****: *p* < 0.001) or comparing BMSC-EV and ADSC EV ($: *p* < 0.05, $$: *p* < 0.01, ns: not statistically significant).

One of the hallmarks of wound healing is cell migration [34] and thus the scratch assay was performed to compare the activity of MSC-EVs in inducing cell migration (Figure 5A). In this case, both BMSC and ADSC-EVs showed the same effect as they promoted the in vitro wound closure and migration of fibroblasts and keratinocytes in a statistically significant manner. Differently to the pro-proliferative effect, both EV types showed a beneficial effect on the migration of endothelial cells, with a higher effect of ADSC-EVs. After observing a different effect on endothelial cell migration, EV ability to induce vessel formation in vitro was directly evaluated (Figure 5B). We demonstrated that both EVs can induce capillary-like structures with a higher statistically significant effect of ADSC-EVs.

Taken together, these data demonstrated that at the dose and timing analyzed, MSC-EVs could play different biological effects on different cell types involved in skin wound healing, including fibroblasts, keratinocytes, and endothelial cells. Both MSC-EVs had the same effect on fibroblast and keratinocyte migration ability, whereas ADSC-EVs were more effective in inducing endothelial cell migration and vessel formation, and BMSC-EVs in promoting proliferation and cell viability.

## 3. Discussion

Diabetes is characterized by an impaired skin wound healing process which leads to the development of chronic ischemic ulcers. This type of lesions reduces physical activity and ambulation and even leads to limb amputation [35]. Both BMSC and ADSC-EVs are known for their regenerative activity on wound healing but, to date, there is no evidence of a comparative analysis of their biological activity on a in vivo diabetes model of skin wound. Here, we directly compared the therapeutic effect of EVs isolated by BMSCs and ADSCs on full-thickness excisional skin wounds on diabetic mice showing that ADSC-EVs were more effective than BMSC-EVs and accelerated wound closure. Bioinformatic analysis of miRNAs and proteins carried on MSC-EVs revealed that the majority of their content was shared by both EVs, but relevant differences in the molecules selectively expressed in ADSC and BMSC-EVs can explain their different biological activity. Finally, the correlation of BMSC and ADSC-EVs to different biological processes was confirmed by in vitro studies. Results showed that ADSC-EVs mainly promoted angiogenesis whereas BMSC-EVs induced cell proliferation on cells involved in skin wound healing.

Our data demonstrated that ADSC-EVs, but not BMSC-EVs, accelerate wound healing in diabetic lesions (Figure 2). The absence of a therapeutic effect of BMSC-EVs in a in vivo diabetic model is consistent with previous observations [21]. Moreover, Pellizzo at al. already showed a higher therapeutic activity of ADSC-EVs in comparison to BMSC-EVs following intradermal injection in a normal skin wound model [36]. Our histological analysis demonstrated an increased epithelial thickness, re-epithelization and number of blood vessels in the wound sites after treatment with ADSC-EVs. Angiogenesis is essential to restore blood flow and promote wound healing of damaged skin [37]. In fact, blood vessels provide nutrients, soluble factors, oxygen, and circulating stem or progenitor cells to the injured sites to sustain tissue regeneration [38]. Our and other groups already demonstrated the essential role of angiogenesis in diabetic skin wound both in vivo [39,40] and in vitro [41]. Consistently with our results, the secretome analysis performed by Nakanishi et al. in 2011 have demonstrated that ADSC secrete significantly large amounts of angiogenic factors [24].

In order to identify differences in the MSC-EV cargo that could explain their different therapeutic effect, the miRNA and protein content of EVs was investigated. The analysis revealed that the majority of miRNAs expressed in MSC-EVs (99 miRNAs) was shared by both EV types (Figure 2A). In fact, Baglio et al. already showed that BMSC and ADSC-EVs have a substantial similarity among the most represented miRNAs [28]. Of note, we found that ADSC-EVs were enriched in miRNAs with respect to BMSC-EVs (Figure 3A,B). The proteomic analysis showed a similar pattern with a relevant part of proteins shared by both EV types (38) and a higher number of proteins selectively expressed in ADSC-EVs (41 proteins) with respect to BMSC-EVs (24 proteins). Despite their similarities, ADSC and BMSC-EVs demonstrated a different biological effect both in vivo and in vitro. In particular, in vitro studies demonstrated that ADSC and BMSC-EVs play regenerative effect on cellular processes. The general beneficial effect of BMSC and ADSC-EVs could be due to their shared cargo. In fact, miRNAs shared by both MSC-EVs were significantly correlated to the EGF receptor axis and its downstream PI3K/Akt signaling pathway, which controls various endothelial cell functions impaired in diabetic wound healing, ranging from migration, to proliferation and to survival [42,43]. This can possibly explain the beneficial effect of both EV types on endothelial cells. Moreover, several miRNAs expressed in both MSC-EVs could explain EV proangiogenic effect including miR-31-5p [44], miR-125a-5p [45], miR-126-3p [18,46]; miR-221-3p [20], miR-130a [47] and miR-132 [48]. In particular, our group already demonstrated the relevance of miR-126 for EV proangiogenic activity showing that EVs derived from ADSCs from obese patients have a reduced proangiogenic ability due to a reduced miR-126 content [49]. Recently, Yu et al. demonstrated that miR-221-3p content is essential for the pro-angiogenic effect of modified BMSC-EVs [20]. Moreover, a recent work of Bi et al. demonstrated that a different miRNA contained in both MSC-EVs, miR-146a, is essential for wound healing [50]. Similarly, the commonly expressed miR-27b accelerates cutaneous wound healing via E3 ubiquitin ligase ITCH [51]. Our data demonstrated that both BMSC and ADSC-EVs promoted fibroblast and keratinocyte migration in vitro (Figure 5A). The ability of EVs to drive keratinocyte and fibroblast migration during wound healing was correlated to their CD73 content [52] and previous reports demonstrated that BMSC and ADSC express similar level of CD73 [7]. Here, we demonstrated that BMSC and ADSC-EVs express similar levels of CD73 by FACS analysis (Figure 1C), possibly explaining their similar effect on keratinocyte and fibroblast migration in diabetic wound healing process. 

Besides their similarities, MSC-EVs showed relevant differences in their activity *in vitro.* In particular, BMSC-EVs mainly promoted cell proliferation, whereas ADSC-EVs exerted a higher induction of endothelial cell migration and angiogenesis. The higher proangiogenic effect of ADSC-EVs was supported by a recent work of Change et al. [53] that showed how EVs isolated from ADSCs exert a significantly higher proangiogenic activity in vitro on HUVECs compared to those isolated from BMSCs. Moreover, we showed that ADSC-EVs, unlike BMSC-EVs, contain additional miRNAs essential for the wound healing process, such as miR-21-3p [54], and for the angiogenesis process, such as miR-210 and miR-378 [55]. Interestingly, bioinformatic analysis of pathways related to the miRNA content revealed that miRNAs carried only by ADSC-EVs, and not by BMSC-EVs, are associated to HIF-1 signaling pathway (Figure 3C). HIF-1 is a key player in the cellular response by modulating expression of genes involved in different cellular processes, including the promotion of angiogenesis [56,57,58]. Additionally, TGFβ signaling pathway was strongly linked to ADSC-EVs. In fact, it was associated to miRNAs specifically expressed by ADSC-EVs and to miRNAs commonly present in both MSC-EVs (Figure 3C). Interestingly, TGFβ signaling cascade has been already documented as a relevant mediator of EV proangiogenic activity, supporting the superior proangiogenic activity of ADSC-EVs [59]. 

Similarly to miRNA analysis, the proteomic profiling showed an enrichment for ADSC-EVs with 38 proteins shared by MSC-EVs, 41 proteins expressed only in ADSC-EVs and 24 proteins present only in BMSC-EVs (Figure 3D). Interestingly, proteins only expressed in ADSC-EVs, such as Wnt, FGF, EGF, PDGF, TGFβ, IL1R1, were associated to signaling pathways related to angiogenesis and to the angiogenesis pathway. The Wnt pathway is associated to the proangiogenic activity of EVs and the activation of endothelial function and healing [60,61]. Moreover, ADSC-EVs were enriched in proteins involved in the activation of angiogenesis during wound healing, including TGFβ, FGF, PDGFR, TNF, and the angiogenesis regulator angiopoietin-1 (ANGPT1) [62,63]. ANGPT1 was only detected in ADSC-EVs and its effect on the rescue of endothelial cell protein permeability could support their biological effect [63]. In addition, IL1R1 was involved in the wound healing ability of MSC-EVs to maintain rapid wound healing in the gingiva via the Fas/Fap-1/Cav-1 cascade [64]. Unlike ADSC-EVs, EVs derived from BMSCs contain proteins linked to integrin and cadherin signaling pathways. Cadherins and integrins are two major families of adhesion molecules involved in essential cellular processes such as adhesion and proliferation [65,66]. In particular, integrins play a relevant role in the regulation of keratinocytes and endothelial cell survival and proliferation [67,68]. Moreover, our analysis demonstrated that BMSC-EVs contained proteins associated to metabolomic processes such as fructose galactose metabolism and glycolysis [69,70]. The fact that proteins only present in BMSC-EVs were mainly associated to adhesion signaling and metabolic pathways could explain their major effect on the proliferation and viability of endothelial cells and keratinocytes.

Overall, our results suggested that the more pronounced proangiogenic activity of ADSC-EVs was essential for accelerating would healing of diabetic wounds, whereas the pro-proliferative effect of BMSC-EVs was insufficient to guarantee a therapeutic effect. Despite keratinocytes and fibroblasts play important roles in the re-epithelization process, collagen synthesis and wound contraction [71], the fact that BMSC-EVs were ineffective in the in vivo model suggests that the angiogenic activation induced by ADSC-EVs was the most relevant event required for diabetic wound healing. This is the first direct evidence showing an advantage of ADSC-EVs over EVs from other MSCs for the treatment of diabetic skin wounds. Although their similarity, ADSC and BMSC-EVs exerted different therapeutic properties or level of activity, suggesting the importance of the EV source for their therapeutic application. 

One limitation of the current study relies on the physiology and structure of rodent skin, which does not completely reflect the human setting or different preclinical models developed in pigs, which more appropriately mimic human injury/repair [72]. Moreover, the aim of this study was to compare the therapeutic effect of BMSC and ADSC-EVs on diabetic skin wounds in the same experimental conditions and at the same dose of EVs. In these conditions, BMSC-EVs did not promote wound healing in a diabetic model. The biological effect is consistent with previous report by Li et al. [21]. We cannot exclude the possibility that higher doses of BMSC-EVs could exert therapeutic effects on similar models [18,19,20].

To date, numerous attempts have been made to accelerate the diabetic wound healing process, but optimal therapeutic strategies are still needed [71]. Here, we showed that ADSC-EVs may promote angiogenesis in the wound sites by increasing the blood vessels density and thereby the wound healing process. Novel therapies to stimulate angiogenesis are highly promising for treating a diabetic wound [20]. In this context, EVs are promising therapeutics as they are not immunogenic, non-tumorigenic, highly stable, easily available at the wound sites and without vascular obstructive concerns [71]. EVs can be easily delivered to the injured site by direct topical application, intradermal injection and integration into biomaterials. Moreover, the pro-angiogenic potential of ADSC-EVs could be increased by using preconditioning of cultured cells as it has already done for BMSC-EVs [18,19,20,21]. For instance, it has been showed that culturing ADSC-EVs in presence of PDGF or under hypoxia can increase the proangiogenic activity of EVs [73,74].

## 4. Materials and Methods

### 4.1. Cell Culture

Human bone marrow MSCs (BMSCs) and adipose MSCs (ADSCs) purchased from Lonza (Basel, Switzerland) were cultured in mesenchymal stem cell basal medium (MSCBM, Lonza, Basel, Switzerland) or ADSC growth medium (ADSCBM, Lonza, Basel, Switzerland), respectively. Cells were cultured for 15 days following thawing from passage one and then after every seven days for successive passages. Cells were used to isolate EVs until passage six for BMSCs and seven for ADSCs and viability after starvation was established around 90% for both cell types (90% ± 2% for BMSC and 92% ± 3% for ADSC). For in vitro experiments, dermal microvascular endothelium cells (HMEC-1) were obtained by the ATCC (Manassas, VA, United States) and cultures following manufacturer’s instruction with MCDB131 medium (Thermo Fisher Scientific, Waltham, MA, USA). Normal Human Dermal Fibroblasts (NHDF) are primary adult fibroblasts (Lonza, Basel, Switzerland). NHDF were cultured in FGM-2 Growth Media (Lonza, Basel, Switzerland) supplemented with bullet kit and 1 mL Mycozap PR (Lonza, Basel, Switzerland). Normal Human Epidermal Keratinocytes (adult skin) (NHEK) were purchased from Lonza and cultured with KGMTM Gold Keratinocyte Growth Medium (Lonza, Basel, Switzerland).

### 4.2. Isolation of EVs

For EV isolation, BMSCs and ADSCs at 70% of confluence were washed several times with PBS (Lonza, Basel, Switzerland) to eliminate traces of serum and then cultured in DMEM (Lonza, Basel, Switzerland) with penicillin/streptomycin and L-glutamine (Sigma-Aldrich, St Louis, MO, USA) without Fetal Bovine Serum (FBS) (Thermo Fisher Scientific Waltham, MA, USA) overnight (16 h) in 5% CO2 incubator at 37 °C. Cell culture supernatants were centrifuged at 4000 rcf for 10 min at 4 °C and submitted to microfiltration using a 0.22 µm vacuum filter unit (Millipore, Burlington, MA, USA) to eliminate cell debris and apoptotic bodies, and then ultracentrifuged twice at 100,000 rcf, for 2 h, at 4 °C in a Beckman Coulter Optima L-90K ultracentrifuge with rotor 45 Ti in polycarbonate tubes (355655; Beckman Coulter, Indianapolis, IN, USA). Pellets were resuspended in PBS (Lonza, Basel, Switzerland) supplemented with 1% DMSO (Sigma-Aldrich, St Louis, MO, USA) and stored at −80 °C for further experiments. In order to take into account donor’s variability, EVs isolated from three donors were used as biological replicates for characterization and in vitro experiments, whereas EVs isolated from MSCs of different donors were pooled together and used for in vivo treatments.

### 4.3. Nanosight Analysis of EVs

EVs were analyzed by nanoparticle tracking analysis (NTA), using the NanoSight LM10 system (NanoSight, Salisbury, UK). The NanoSight system was equipped with a 405 nm laser and NTA 3.1 analytic software. The analysis allowed to define EV concentration and size profile. The Brownian movements of EVs were subjected to a laser light source and were recorded by a camera. The analytic software converted this information into size and concentration parameters using the Stokes-Einstein equation. For each sample, three videos of 30 s duration were recorded, and camera levels were set for all the acquisition at 15. Briefly, EVs were diluted 1:200 in 1 mL of saline solution (Fresenius Kabi, Bad Homburg vor der Höhe, Germany) previously filtered with 0.22 µm membranes (Millipore, Burlington, MA, USA). NTA post-acquisition settings were optimized and maintained constant among all samples, and each video was then analyzed to measure EV size mean and concentration.

### 4.4. Transmission Electron Microscopy Analysis of EVs

EVs were analyzed using transmission electron microscopy analysis. For this, fresh EV preparations were placed on 200 mesh nickel formvar carbon-coated grids (Electron Microscopy Science, Hatfield, PA, USA) and left to adhere for 20 min. Next, grids were incubated with 2.5% glutaraldehyde containing 2% sucrose. After washing in distilled water, samples were negatively stained with Nano-W and NanoVan (Nanoprobes, Yaphank, NY, USA) and analyzed using a Jeol JEM 1010 electron microscope (Jeol, Tokyo, Japan) (Department of Neuroscience, University of Turin) [75].

### 4.5. FACS Characterization of EVs

EVs were characterized by cytofluorimetric analysis using the following fluorescein isothiocyanate (FITC) or phycoerythrin (PE) conjugated antibodies: CD73, CD105, CD44 (Miltenyi Biotec, Bergisch Gladbach, Germany). Conjugated mouse non-immune isotypic immunoglobulin G (IgG) (Miltenyi Biotec, Bergisch Gladbach, Germany) was used as control. Briefly, 10 µL of EVs were labeled for 15 min at 4 °C with antibodies and immediately diluted 1:3 and acquired [76].

For analysis using the human cytofluorimetric bead-based MACSPlex exosome kit (Miltenyi Biotec, Bergisch Gladbach, Germany) the manufacturer’s protocol was followed [77]. Briefly, EV preparations consisting of approximately 1 × 10^9^ EVs/preparation were diluted inMACSPlex buffer (MPB) to a final volume of 120 µL in a 1.5 mL microcentrifuge tube. Then, 15 µL of MACSPlex exosome capture beads (containing a cocktail of 39 different exosomal marker epitopes) was added at each sample. Samples counterstained by adding 15 µL of mix of APC-conjugated antibody containing anti-CD9, anti-CD63, and anti-CD81 and incubated overnight at room temperature in the dark on an orbital shaker at 450 rpm. Post incubation, beads were washed with 1 mL of MPB at 3000 g for 5 min, followed by a longer washing step by incubating the beads in 1 mL of MPB on an orbital shaker for 15 min. Then, samples were centrifuged at 3000× *g* for 5 min and the supernatant was aspirated leaving a residual volume of 150 µL per tube for acquisition. During acquisition, the median fluorescence intensity (MFI) for all 39 exosomal markers were corrected for medium background and gated based on their respective fluorescence intensity as per manufacturer’s instructions. All cytofluorimetric analysis were performed using the CytoFLEX flow cytometer (Beckman Coulter, Indianapolis, IN, USA) equipped with the CytExpert software version 2.3.0.84. For both classical FACS and MACSPlex exosome kit each analysis include 6 biological replicates.

### 4.6. Protein Measurement and Western Blot Analysis

Either ADSC and BMSC-EVs and cells were homogenized in RIPA buffer (Millipore Sigma, St Louis, MO, USA) supplemented with a cocktail of protease (Millipore Sigma) and phosphatase (Millipore Sigma, St Louis, MO, USA) inhibitors and phenylmethylsulphonyl fluoride (PMSF, Millipore Sigma, St Louis, MO, USA). Protein concentration was measured by bicinchoninic acid (BCA) protein assay (Pierce, Thermo Fisher Scientific, Waltham, MA, USA) following manufacturer’s instruction. Briefly, 10 µL of sample were dispensed into wells of a 96-well plate and total protein concentrations were determined using a linear standard curve established with BSA. Then, 5 μg of protein from EV and cell lysate were mixed with 6 µL of Laemmli buffer (BioRad, Hercules, CA, USA) with β-mercaptoethanol (Millipore Sigma, St Louis, MO, USA) and PBS was added to reach the equal volume of 40 µL. Aliquots were denatured for 5 min at 95 °C in a thermomixer then cooled in ice and loaded onto 4–20% Criterion TGX Stain-Free Precast gels (Bio-Rad, Hercules, CA, USA) and transferred onto nitrocellulose membranes (BioRad, Hercules, CA, USA). Membranes were blocked with 5% nonfat milk in PBS containing 0.1% Tween 20 (PBS-T) for one hour at RT, and then incubated in primary antibodies overnight at 4 °C. The next day, membranes were washed three times with PBS-T and incubated in species-specific, horseradish peroxidase (HRP)-conjugated secondary antibodies for two hours at RT. Membranes were washed three times with PBS-T prior to the addition of the chemiluminescent substrate SuperSignal West Femto Maximum Sensitivity Substrate (Thermo Fisher Scientific). Chemiluminescent protein detection was visualized with the ChemiDoc™ MP Imaging System (BioRad, Hercules, CA, USA). The following primary antibodies were used: anti-CD29/ITGb1 (1:500; Invitrogen, Carlsbad, CA, USA), anti-CD9 (1:1000, Invitrogen, Carlsbad, CA, USA), anti-CD63 (1:1000, Invitrogen, Carlsbad, CA, USA), anti-CD81 (1:1000, Invitrogen, Carlsbad, CA, USA), anti-Alix (1:200; SantaCruz, Santa Cruz CA, USA), anti-GM130 (1:1000, abCam, Cambridge, UK), anti-RS29 (1:1000, abCam, Cambridge, UK). The following HRP-conjugated secondary antibodies were used: Goat anti-Rabbit (1:5000, Pierce, Thermo Fisher Scientific, Waltham, MA, USA), Goat anti-Mouse (1:5000, Pierce, Thermo Fisher Scientific, Waltham, MA, USA).

### 4.7. Single Particle Interferometric Reflectance Imaging Sensor with ExoView^®^

ADSC and BMSC-EVs were diluted with Incubation Solution (NanoView Biosciences, Brighton, MA, USA) at the final concentration of 1 × 10^8^ EVs. Then, 35 μL of the sample were carefully pipetted onto the silicon custom chip coated with individual antibody spots against human CD9, CD63, CD81, CD105 and CD44 as well as negative isotype controls. After overnight incubation in a 24-well plate, chips were processed according to manufacturer’s instructions. Briefly, the chips were washed in 750 μL of incubation buffer for 3 min three times on an orbital shaker, then incubated for one hour at RT with a cocktail of fluorescent antibodies, anti-CD81 (green), anti-CD63 (blue), and anti-CD9 (red), provided by NanoView Biosciences, Brighton, MA, USA). Chips were washed once in Incubation Solution, three times in Wash Solution, and once in Rinse Solution. Chips were carefully removed from the 24-well plate, washed further in deionized water and removed for drying. Image and data acquisition for each chip was performed with the ExoView^®^ R100 (NanoView Biosciences, Brighton, MA, USA) machine and nScan acquisition software (NanoView Biosciences, Brighton, MA, USA). The reader automatically acquires interferometric and fluorescence emittance images of the chip. Data analysis was performed with NanoViewer 2.9.3 (NanoView Biosciences, Brighton, MA, USA) [78]. Analysis was performed using four chips for each type of EV with three spots for each capturing antibody in a single chip.

### 4.8. Diabetic Wound Healing Mouse Model

Animal studies were conducted in accordance with National Institute of Health Guidelines for the Care and Use of Laboratory Animals. All procedures were approved by the Ethics Committee of the University of Turin and the Italian Health Ministry (authorization number: 739/2016-PR). Male NSG mice of eight-week-old were purchased from the animal facility at the Molecular Biotechnology Centre (MBC, Turin, Italy). Intraperitoneal injection of streptozotocin (STZ) (42 mg/kg) were used to induce diabetes. For that STZ was dissolved in freshly made 0.1 mol/L citrate buffer, at pH 4.5, for four consecutive days in order to avoid acute STZ toxicity [79]. The onset of diabetes was established by measuring glycaemia (up to 200 mg/mL) 12 days after the last STZ injection using a blood glucometer (GlucoMen LX Plus+, A. Menarini Diagnostics, Florence, Italy). Five diabetic animals for each group were anesthetized and back cutaneous hair was removed by electrical shaving. Four 6-mm diameter full-thickness skin wounds were created on each side of the midline using a disposable biopsy punch (PMD Medical, Nîmes, France). Each animal received different treatments on wounds: carboxymethylcellulose high viscosity 10 mg/mL (Sigma-Aldrich, St Louis, MO, USA) (vehicle) or carboxymethylcellulose and BMSC (BMSC-EVs) or ADSC-EVs (ADSC-EVs). Each treatment was performed using 25 µL of vehicle and 1 × 10^9^ EVs. The day of the wounding was counted as day 0. Animals were monitored at different times (0, 3, 7, 10 days for mice treated with BMSC-EVs and 0, 3, 7, 10, 14 days for mice treated with ADSC-EVs) by wound measurement. At each time, the wounds received a fresh application of the treatments. At the last timepoint, mice were sacrificed and back skin wounds were collected for histological analysis. Histological analysis was performed using hematoxylin and eosin (H&E) staining and was used to measure the scar width, the percentage of re-epithelization as the percentage of newly formed epithelium in the wound and the epithelial thickness of the wounds, using ImageJ software (NIH, Bethesda, MD, USA). The number of vessels was evaluated by counting the vessels per field in 3 randomly chosen sections of skin (original magnification 40×) for each wound section using ImageJ software version 1.49v (NIH).

For evaluation of skin wound healing in a not-diabetic model, four adult male Balb/c mice (6–8 weeks) were purchased from the animal facility at the Molecular Biotechnology Centre (MBC, Turin, Itay). Full-thickness skin wounds were made as for diabetic mice and the wound are was monitored at different times (0, 3, 7 days) by wound measurement. 

### 4.9. In Vitro Proliferation Assay

Cells (endothelial cells, keratinocytes, and fibroblasts) were seeded at 2000 cells/well into 96-well plates with their culture medium and left to adhere. After 6 h, cells were washed with PBS (Lonza, Basel, Switzerland) and cell medium was replaced with DMEM (Euroclone, Pero, MI, Italy) without the addition of FBS (Thermo Fisher Scientific, Waltham, MA, USA) for an overnight starvation. After 14 h, cells were treated with following stimuli: DMEM 0% FBS as negative control for untreated cells (NT), DMEM 0% FBS with 50,000 EVs/cell for BMSC or ADSC-EVs (BMSC or ADSC-EV) and different positive controls (CTR+) for each cell type (MCDB131 medium for endothelial cells, FGM-2 Growth Media for fibroblasts and KGMTM Gold Keratinocyte Growth Medium for keratinocytes). DNA synthesis was detected as incorporation of 5-bromo-2′-deoxyuridine (BrdU) into the cellular DNA using an ELISA kit (Roche, Basel, Switzerland), following the manufacturer’s instructions. Briefly, BrdU was added to culture conditions for 12 h and the acquisition was performed after 24 h of treatment with EVs. Optical density was measured with an ELISA reader (Bio-Rad, Hercules, CA, USA). Three experiments were performed in triplicate and results are expressed as ratio in comparison to untreated cells (NT).

### 4.10. In Vitro Viability Assay

Cells (endothelial cells, keratinocytes, and fibroblasts) were seeded at 2000 cells/well into 96-well plates with their culture medium and left to adhere. After 6 h, cells were washed with PBS (Lonza, Basel, Switzerland) and cell medium was replaced with DMEM (Euroclone, Pero, MI, Italy) without the addition of FBS (Thermo Fisher Scientific, Waltham, MA, USA) for an overnight starvation. After 14 h, cells were treated with following stimuli: DMEM 0% FBS as negative control for untreated cells (NT), DMEM 0% FBS with 50,000 EVs/cell for BMSC or ADSC-EVs (BMSC or ADSC-EV) and different positive controls (CTR+) for each cell type (MCDB131 medium for endothelial cells, FGM-2 Growth Media for fibroblasts and KGMTM Gold Keratinocyte Growth Medium for keratinocytes). After 24 h the reagent AlamarBlue was added to each well following manufacturer’s instruction (Thermo Fisher Scientific, Waltham, MA, USA) and viability was measured 48 h after EV treatment with an ELISA reader (Bio-Rad, Hercules, CA, USA). Three experiments were performed in triplicate and viability was calculated as percentage in comparison to untreated cells (NT).

### 4.11. In Vitro Scratch Test

Cells (endothelial cells, keratinocytes, and fibroblasts) were seeded at a density of about 5 × 10^3^ cells/well in 24-well plates in the appropriate culture medium. When the cells reached complete confluence, scratch were created with a sterile tip to mimic a wound. Prior to stimulation (t = 0), micrographs of the well were obtained using a Leica camera (Leica, Wetzlar, Germany). The cells were then stimulated with DMEM alone (untreated cells, NT), BMSC or ADSC-EVs 50,000 EVs/cell in DMEM (BMSC or ADSC-EV) or with positive controls (CTR+): MCDB131 medium for endothelial cells, FGM-2 Growth Media for fibroblasts and KGMTM Gold Keratinocyte Growth Medium for keratinocytes. The ‘wound closure’ phenomenon was monitored for 24 h, taking pictures using the Leica camera. Images were analyzed by ImageJ software (NIH) measuring the wound area. Three experiments were performed in triplicate and results are expressed as a percentage of wound closure considering the wound area at t = 0 as 100% and calculating the corresponding area occupied by cells at 24 h. 

### 4.12. In Vitro Formation of Capillary-Like Structures Assay

To evaluate the formation of capillary-like structures, HMEC were seeded at 20,000 cells/well into 24-well plate precoated with growth factor-reduced Matrigel (Corning, Tewksbury, MA, USA). Cells were treated with DMEM 0% FBS as negative control for untreated cells (NT), DMEM 0% FBS with 50,000 EVs/cell for BMSC or ADSC-EVs (BMSC or ADSC-EV) and EndoGRO-VEGF Complete Culture Media (Millipore, St Louis, MO, USA) (CTR1+) or DMEM 0% FBS plus 0.1 µg/mL EGF (Sigma-Aldrich, St Louis, MO, USA) (CTR2+) as positive controls. After 24 h, cells were observed with a Nikon-inverted microscope (10×), and photographed with a Leica-digital camera (five photos for each well, in triplicate). Image analysis was performed automatically with the Angiogenesis Analyzer tool by ImageJ 1.49v software (NIH). Three experiments were performed in triplicate and results are expressed as ratio in comparison to the untreated cells (NT).

### 4.13. miRNA Expression Analysis 

miRNA expression analysis was performed by qRT-PCR array on three different samples of BMSC-EVs. Briefly, RNA was extracted from purified EVs by mirVana Isolation Kit (Thermo Fisher Scientific, Waltham, MA, USA), according with manufacturer’s instruction. RNA concentration was measured by Nanodrop ND-1000 (Thermo Fisher Scientific, Waltham, MA, USA), and the ratio 260/280 and 260/230 showed absence of contaminants. Fifty nanograms of total RNA were retro-transcribed to cDNA with TaqMan^®^ MicroRNA Reverse Transcription Kit (Thermo Fisher Scientific, Waltham, MA, USA). cDNA was pre-amplified with Megaplex™ RT Primers, Human Pool Set v3.0 and TaqMan^®^ PreAmp Master Mix (Thermo Fisher Scientific, Waltham, MA, USA) by using Veriti Thermal Cycler (Thermo Fisher Scientific, Waltham, MA, USA). The expression profile of a panel of 754 human miRNAs was evaluated by TaqMan^®^ Array Human MicroRNA Card Set v3.0 (Thermo Fisher Scientific, Waltham, MA, USA) with QuantStudio12k Flex system (Thermo Fisher Scientific, Waltham, MA, USA). ExpressionSuite Software 1.1 (Thermo Fisher Scientific, Waltham, MA, USA) was used to calculate Ct values. Values of Amp score < 1.1 or Cq conf < 0.7 or Ct > 34 were excluded from analysis. Only miRNAs expressed in all samples of the same group were included for analysis.

### 4.14. Target Pathway and Enrichment Analysis

For miRNA and protein content evaluation a bioinformatic analysis was performed. For protein analysis, protein dataset already published were used to compare BMSC-EVs [30] and ADSC-EVs [31] cargo. For miRNA analysis, the bioinformatic analysis of EV content was performed comparing published ADSC-EV data [32] with expressly prepared BMSC-EV data. For all investigation, three batches of EVs for each source were compared and the same technique was used: TaqMan^®^ Array Human MicroRNA Card for miRNA and Human Antibody Array 1000 kit (RayBiotech, Peachtree Corners, GA, USA) for proteins. The Venn diagrams comparing miRNAs and proteins carried by ADSC- and BMSC-EV were generated with FunRich 3.1.3 software [80]. The lists of miRNAs carried by either ADSC and BMSC-EVs, miRNAs exclusively carried by ADSC-EVs and miRNAs exclusively carried by BMSC-EVs were exported and used for further analysis. The heatmap was generated by Heatmapper online tool (with last access on 20th February 2021) (http://www.heatmapper.ca/) [81] based on Ct values of the list of miRNAs carried by either ADSC and BMSC-EVs. Target pathway analysis was performed for the three lists of miRNA by DIANA-mirPath v.3 [33] online software. miRNA targets were searched on microT-CDS with p-value threshold of 0.05, microT threshold of 0.8, with False Discovery Rate (FDR) correction. Results were merged by “genes-union” criteria. The lists of Kyoto Encyclopedia of Gene and Genomes (KEGG) pathway significantly targeted by the miRNAs provided by the analysis is shown in supplementary materials (Tables S2 and S3). The protein content of EVs was analyzed by the Gene List Analysis tool of Panther online software [82,83] in order to find the signaling pathways targeted by EVs.

### 4.15. Statistical Analysis

Data were analyzed using GraphPad Prism 6.0 Demo (GraphPad, San Diego, CA, USA). Statistical analyses of three or more groups of data were performed using ANOVA with Dunnett’s or Turkey’s multiple-comparisons test as appropriate. T test was used to compare two groups of data. Values were expressed as their mean ± SD. Statistical significance was established at *p* < 0.05 (* or $: *p* < 0.05, ** or $$: *p* < 0.01, *** or $$$: *p* < 0.005, and **** or $$$$: *p* < 0.001).

## 5. Conclusions

Overall, in this study we demonstrated that ADSC-EVs had a more potent proangiogenic activity in comparison to BMSC-EVs and can accelerate the wound healing of diabetic ulcers. This result highlights the opportunity to implement current therapeutic treatments for diabetic skin wounds with proangiogenic factors, such as ADSC-EVs, in order to improve their clinical efficacy. Finally, our data confirmed the importance of the choice of MSC source for EV based on the clinical application desired in order to optimize the therapeutic efficacy.

## Figures and Tables

**Table 1:** List of miRNAs Expressed in BMSC and ADSC-EVs. miRNAs were divided in three groups accordingly to their common expression in both MSC-EVs (Common), expression only in BMSC-EVs (BMSC-EV) or ADSC-EVs (ADSC-EV).

Common	BMSC-EV	ADSC-EV
hsa-let-7c-5p	hsa-let-7a-5p	hsa-miR-10a-5p
hsa-let-7d-5p	hsa-let-7e-5p	hsa-miR-1226-5p
hsa-let-7g-5p	hsa-miR-10b-5p	hsa-miR-125b-1-3p
hsa-miR-100-5p	hsa-miR-130b-3p	hsa-miR-126-5p
hsa-miR-106a-5p	hsa-miR-197-3p	hsa-miR-1270
hsa-miR-106b-5p	hsa-miR-199a-3p	hsa-miR-1291
hsa-miR-10b-3p	hsa-miR-29b-3p	hsa-miR-129-2-3p
hsa-miR-125a-3p	hsa-miR-342-3p	hsa-miR-136-3p
hsa-miR-125a-5p	hsa-miR-365a-3p	hsa-miR-137
hsa-miR-125b-5p	hsa-miR-365b-3p	hsa-miR-140-5p
hsa-miR-126-3p	hsa-miR-483-5p	hsa-miR-142-3p
hsa-miR-1271-5p	hsa-miR-484	hsa-miR-144-5p
hsa-miR-127-3p	hsa-miR-485-3p	hsa-miR-145-3p
hsa-miR-1290	hsa-miR-99a-5p	hsa-miR-148a-3p
hsa-miR-130a-3p		hsa-miR-148b-5p
hsa-miR-132-3p		hsa-miR-149-5p
hsa-miR-133a-3p		hsa-miR-181a-2-3p
hsa-miR-138-5p		hsa-miR-184
hsa-miR-139-5p		hsa-miR-18a-3p
hsa-miR-143-3p		hsa-miR-18a-5p
hsa-miR-145-5p		hsa-miR-193b-5p
hsa-miR-146a-5p		hsa-miR-199-3p
hsa-miR-146b-5p		hsa-miR-19b-1-5p
hsa-miR-150-5p		hsa-miR-203a-3p
hsa-miR-151a-3p		hsa-miR-204-5p
hsa-miR-151a-5p		hsa-miR-210-3p
hsa-miR-152-3p		hsa-miR-21-3p
hsa-miR-155-5p		hsa-miR-214-5p
hsa-miR-15b-5p		hsa-miR-222-5p
hsa-miR-16-5p		hsa-miR-26a-1-3p
hsa-miR-17-5p		hsa-miR-27a-5p
hsa-miR-186-5p		hsa-miR-30a-5p
hsa-miR-191-5p		hsa-miR-31-3p
hsa-miR-192-5p		hsa-miR-320b
hsa-miR-193a-3p		hsa-miR-335-3p
hsa-miR-193a-5p		hsa-miR-339-3p
hsa-miR-193b-3p		hsa-miR-34a-3p
hsa-miR-195-5p		hsa-miR-362-5p
hsa-miR-196b-5p		hsa-miR-374b-5p
hsa-miR-19a-3p		hsa-miR-378
hsa-miR-19b-3p		hsa-miR-379-5p
hsa-miR-20a-5p		hsa-miR-382-5p
hsa-miR-20b-5p		hsa-miR-410-3p
hsa-miR-212-3p		hsa-miR-424-3p
hsa-miR-214-3p		hsa-miR-424-5p
hsa-miR-21-5p		hsa-miR-425-3p
hsa-miR-218-5p		hsa-miR-451a
hsa-miR-221-3p		hsa-miR-452-5p
hsa-miR-222-3p		hsa-miR-455-5p
hsa-miR-223-3p		hsa-miR-495-3p
hsa-miR-224-5p		hsa-miR-505-5p
hsa-miR-24-3p		hsa-miR-542-5p
hsa-miR-25-3p		hsa-miR-543
hsa-miR-26a-5p		hsa-miR-548c-3p
hsa-miR-26b-5p		hsa-miR-590-3p
hsa-miR-27a-3p		hsa-miR-590-5p
hsa-miR-27b-3p		hsa-miR-597-5p
hsa-miR-28-3p		hsa-miR-603
hsa-miR-28-5p		hsa-miR-628-3p
hsa-miR-29a-3p		hsa-miR-628-5p
hsa-miR-301a-3p		hsa-miR-629-3p
hsa-miR-30a-3p		hsa-miR-652-3p
hsa-miR-30b-5p		hsa-miR-655-3p
hsa-miR-30c-5p		hsa-miR-660-5p
hsa-miR-30e-3p		hsa-miR-664a-3p
hsa-miR-31-5p		hsa-miR-7-1-3p
hsa-miR-320a		hsa-miR-766-3p
hsa-miR-323a-3p		hsa-miR-886-3p
hsa-miR-324-3p		hsa-miR-889-3p
hsa-miR-324-5p		hsa-miR-93-5p
hsa-miR-328-3p		
hsa-miR-331-3p		
hsa-miR-335-5p		
hsa-miR-345-5p		
hsa-miR-34a-5p		
hsa-miR-34b-3p		
hsa-miR-370-3p		
hsa-miR-374a-5p		
hsa-miR-376a-3p		
hsa-miR-376c-3p		
hsa-miR-409-3p		
hsa-miR-411-5p		
hsa-miR-432-5p		
hsa-miR-433-3p		
hsa-miR-454-3p		
hsa-miR-487b-3p		
hsa-miR-493-3p		
hsa-miR-494-3p		
hsa-miR-532-3p		
hsa-miR-532-5p		
hsa-miR-539-5p		
hsa-miR-574-3p		
hsa-miR-625-3p		
hsa-miR-708-5p		
hsa-miR-744-5p		
hsa-miR-886-5p		
hsa-miR-92a-3p		
hsa-miR-93-3p		
hsa-miR-99b-3p		

**Table 2:** List of Proteins Expressed in BMSC and ADSC-EVs. Proteins were indicated with their gene symbol and divided in three groups accordingly to their common expression in both MSC-EVs (Common), expression only in BMSC-EVs (BMSC-EV) or ADSC-EVs (ADSC-EV).

Common	BMSC-EV	ADSC-EV
ALDOC	A1BG	ADGRB1
APOC3	ALDOA	ALPP
CNTF	APOA4	ANGPT1
CNTFR	CCL2	BMP5
CSF2	CKM	BMP7
CXCL8	CLUL1	C2
DEFB1	CSF3	CCHCR1
ENPP2	CXCL1	CCL19
F13A1	CXCL9	CCL28
GDF11	EPO	CCL4
GDF3	FN1	CHI3L1
GDF5	IL10	CKB
GDF9	LYN	CLU
GH1	MAN2B1	CSF2RA
GREM1	MINA	CXCL2
GZMA	MUSK	DKK4
IFNG	NPTX1	EPX
IL13	NRG3	FGF10
IL15	PDGFRA	FGF16
IL1A	POMC	FGF18
IL1RL2	RET	GDF1
IL2	S100A8	GFRA3
IL21	TEC	HRG
IL5	THBS1	IAPP
IL6		IL1R1
IL7		IL1RL1
INSR		IL2RB
KRT19		IL9
LALBA		LAG3
MET		LHCGR
MSTN		LTA
S1PR1		MMP20
SERPING1		MSX1
SLC2A5		MUC16
TFRC		PDGFRB
TGFB1		SCGB2A2
TNK2		TNF
TXNIP		TNFRSF13C
		TNFRSF8
		TNNC1
		UBB

## Data Availability

All data are present in the manuscript, there are not link to publicly archived datasets.

## Supplementary Materials

The following are available online at https://www.mdpi.com/article/10.3390/ijms22083851/s1, Figure S1: Western Bolt Membranes of BMSC and ADSC-EV Characterization., Figure S2: Protein content of BMSC and ADSC-EVs, Figure S3: Characterization of ADSC and BMSC-EVs by ExoView®, Figure S4: Comparison of Skin Wound Healing on Normal and Diabetic Ulcers, Figure S5: Hystological analysis of blood vessels in diabetic mice treated with ADSC-EVs, Table S1: miRNAs Carried by ADSC-EVs and MSC-EVs, Table S2: Target Pathways of miRNAs Carried Only by ADSC-EVs, Table S3: Target Pathways of miRNAs Carried by ADSC-EVs and BMSC-EVs, Table S4: Target Pathways of miRNAs Carried Only by BMSC-EVs.
