# Supplementary material for: Differential Therapeutic Effect of Extracellular Vesicles Derived by Bone Marrow and Adipose Mesenchymal Stem Cells on Wound Healing of Diabetic Ulcers and Correlation to Their Cargoes
Source: Int J Mol Sci. 2021 Apr 8;22(8):3851. doi: 10.3390/ijms22083851 (PMC8068154; doi:10.3390/ijms22083851)
Supplement: Supplementary file 1 [file ijms-22-03851-s001.pdf]

International Journal of
Molecular Sciences

MDPI

Supplementary Materials

Article

Differential Therapeutic Effect of Extracellular Vesicles Derived by Bone Marrow and Adipose Mesenchymal Stem Cells on Wound Healing of Diabetic Ulcers and Correlation to Their Cargoes.

Margherita A. C. Pomatto [1], Chiara Gai [1], Federica Negro [2], Massimo Cedrino[3], Cristina Grange[1], Elena Ceccotti[1], Gabriele Togliatto[1], Federica Collino[4], Federico Figliolini[3], Tatiana Lopatina[1], Maria Felice Brizzi[1], Giovanni Camussi[1]*.

International Journal of
Molecular Sciences

MDPI

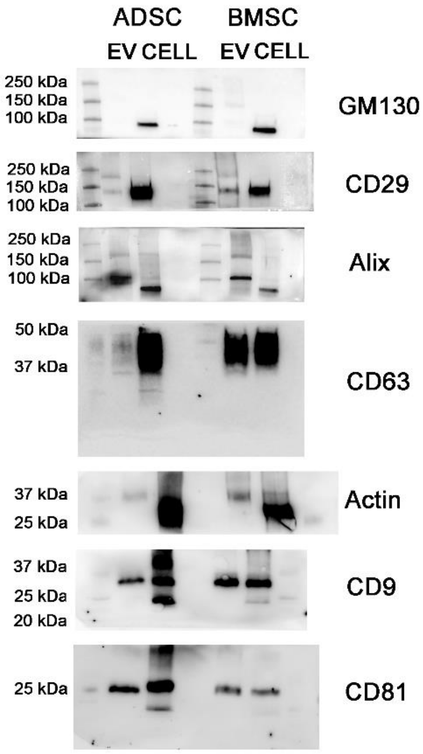

Figure S1. Western Bolt Membranes of BMSC and ADSC-EV Characterization. Images of western blot membranes used in Figure 1 to show MSC-EV characterization.

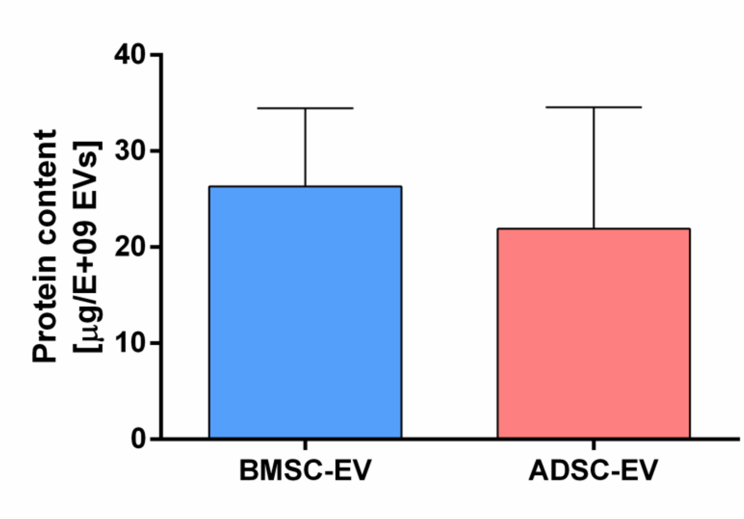

Figure S2. Protein content of BMSC and ADSC-EVs. Quantitative analysis of total proteins contained in BMSC and ADSC-EVs. Proteins were isolated by EV preparation and measured using BCA assay as described in the 'Materials and Methods' section. Data are expressed as mean ± standard deviation.

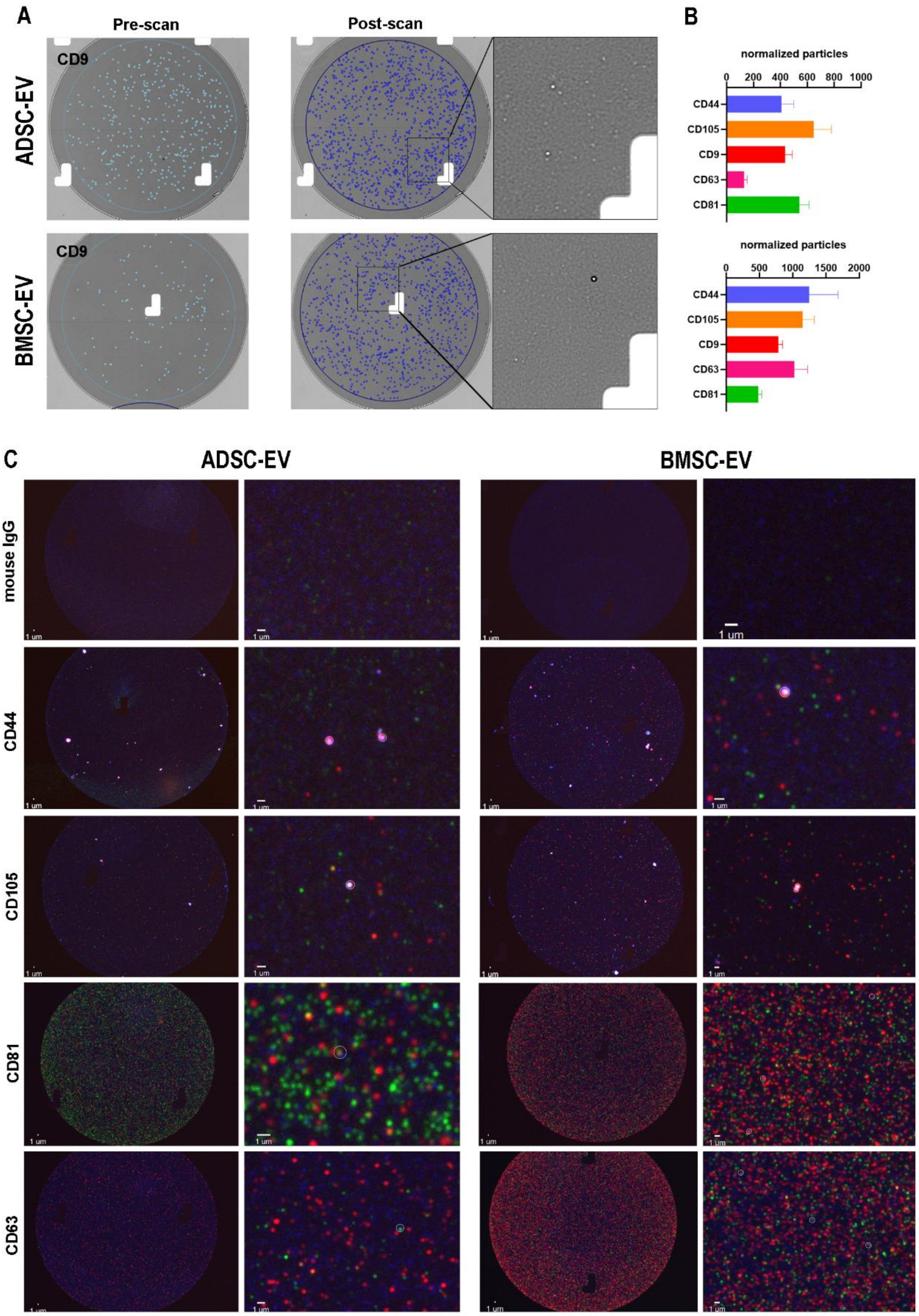

Figure S3. Characterization of ADSC and BMSC-EVs by ExoView®. (A) Interferometry images of a representative anti-CD9 capture spot pre-scan (left) and post-scan (right) for ADSC-EVs (first row) and BMSC-EVs (second row). Blue circles indicate EVs detected by interferometry. A magnified image of post-scan spot

is shown on the right; (B) The histograms show the number of normalized particles counted by interferometry for each capturing antibody. Bars show mean ± SD of 4 chips for each type of EV, each chip contains three spots for each capturing antibody; (C) Representative images for EV detection by fluorescence for ADSC-EVs (left columns) and BMSC-EVs (right columns). Images show capture spot with anti-mouse IgG, anti-CD44, anti-CD105, anti-CD81, anti-CD63. For each EV groups, images on the left show a magnified field of the image on the right. White circles highlight single EV particle stained with either CD9 (red), CD63 (blue), and CD81 (green) confirming the colocalization of the three markers on the same EV. Scale bars of 1 μm are shown on each image.

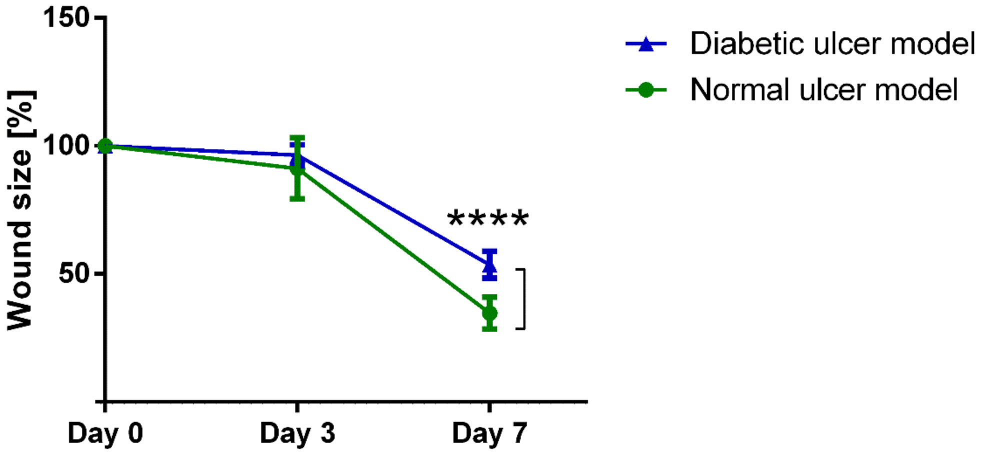

Figure S4. Comparison of Skin Wound Healing on Normal and Diabetic Ulcers. Quantitative analysis of wound size in models of ulcers in normal and diabetic mice. Full-thickness excisional wounds were made at day 0 and wound area were measured at 3 and 7 days and expressed as percentage in comparison to inizial area at day 0. ****: p<0.0001 between normal and diabetic model at day 7.

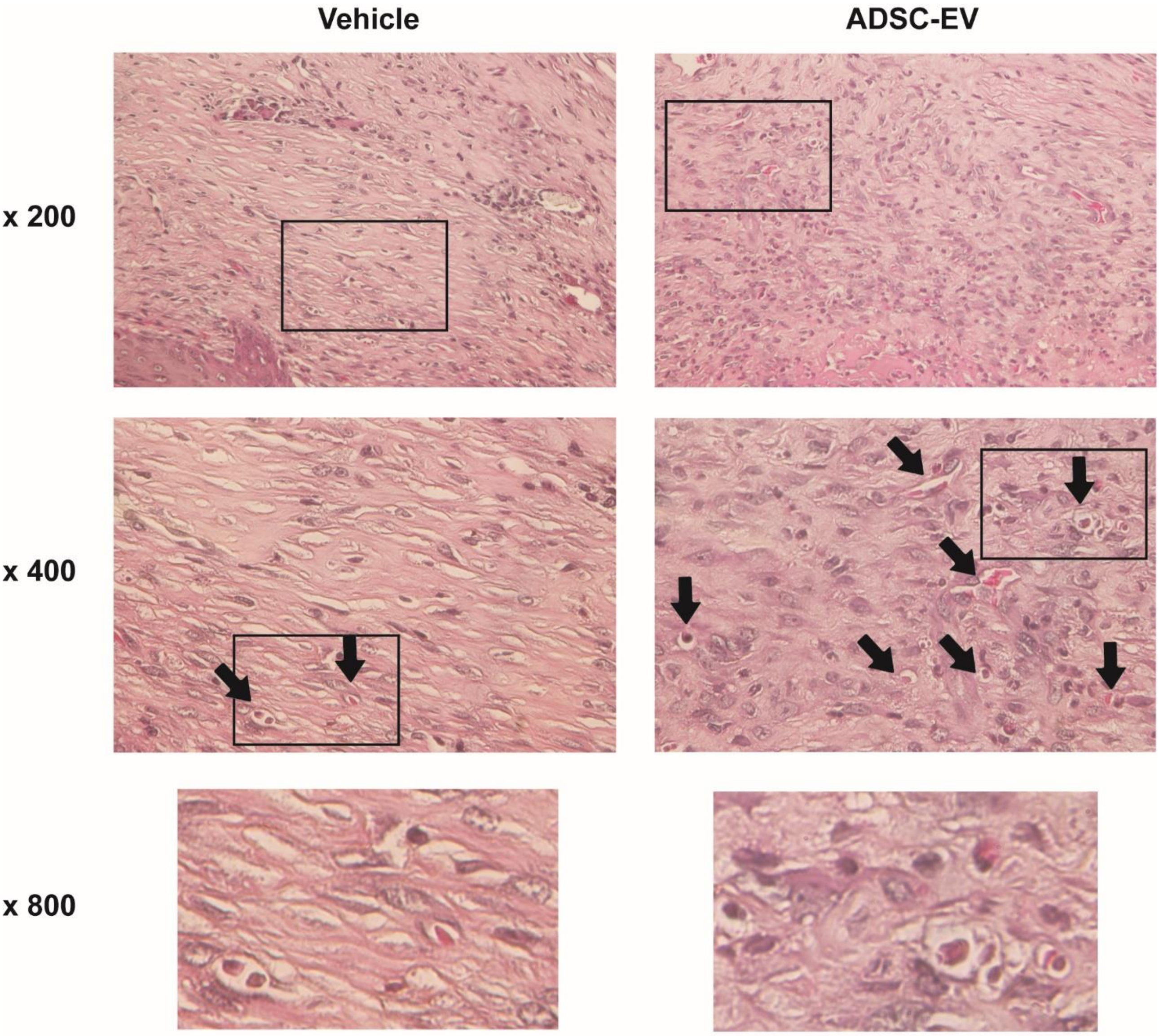

Figure S5. Hystological analysis of blood vessels in diabetic mice treated with ADSC-EVs. Representative images of H&E staining at different magnification (x 200, x 400 and x 800) and detection of vessels present in wound sections. Black arrows indicate vessels in the micrograph. Black rectangles indicate the site of magnification.

International Journal of Molecular Sciences

MDPI

Table S1: miRNAs Carried by ADSC-EVs and MSC-EVs.

miRNA expression levels detected by qRT-PCR in three EV samples from ADSC and BMSC-EVs. Data were obtained by human TaqMan® Array Human MicroRNA Card and are expressed as cycle threshold (Ct). Absence of Ct value indicates that miRNA was not expressed in the sample.

miRNA	BMSC-EV 1	BMSC-EV 2	BMSC-EV 3	ADSC-EV 1	ADSC-EV 2	ADSC-EV 3
let-7a-5p	28.010	26.042	26.716			
let-7c-5p	29.357	30.271	28.879	27.012	25.964	30.417
let-7d-5p	28.381	28.931	27.586	25.129	25.124	29.065
let-7e-5p	25.824	25.565	24.891			
let-7g-5p	29.336	29.764	28.563	26.482	24.586	29.971
miR-100-5p	24.947	25.048	22.918	22.775	19.867	25.311
miR-106a-5p	26.956	27.992	26.442	21.770	23.377	25.118
miR-106b-5p	29.702	29.955	29.877	25.399	25.927	27.738
miR-10a-5p				25.952	25.369	30.766
miR-10b-3p	30.959	30.292	33.630	25.866	29.698	26.352
miR-10b-5p	28.433	31.291	29.856			
miR-1226-5p				30.028	29.179	30.789
miR-125a-3p	33.424	33.675	32.509	30.804	29.210	31.244
miR-125a-5p	27.662	29.406	25.085	24.838	22.818	27.947
miR-125b-1-3p				30.380	28.051	31.541
miR-125b-5p	25.195	25.779	23.281	23.298	22.462	26.491
miR-126-3p	29.696	29.390	29.455	20.864	24.166	25.699
miR-126-5p				26.292	30.122	32.689
miR-1270				32.701	30.497	32.582
miR-1271-5p	32.612	30.090	29.748	28.913	27.187	27.398
miR-127-3p	30.474	30.089	27.003	25.713	24.063	28.566
miR-1274A	26.987	22.658	21.300	17.450	16.030	21.196
miR-1274B	26.721	21.940	21.171	15.819	15.850	19.979
miR-1290	33.263	29.771	30.809	22.933	24.103	26.371
miR-1291				32.474	26.652	30.451
miR-129-2-3p				31.401	30.392	33.448
miR-130a-3p	32.008	32.981	30.524	28.533	26.044	29.708
miR-130b-3p	31.990	32.052	31.056			
miR-132-3p	27.676	27.702	26.930	24.960	22.728	27.760
miR-133a-3p	31.287	31.646	30.664	27.662	27.934	26.115
miR-136-3p				30.545	28.744	30.956
miR-137				31.652	30.099	32.526
miR-138-5p	29.473	30.618	27.420	27.050	26.944	26.608
miR-139-5p	33.433	32.566	30.290	25.225	29.435	29.715
miR-140-5p				25.345	24.372	28.969
miR-142-3p				25.392	29.673	30.390
miR-143-3p	28.254	28.874	28.099	27.065	25.474	29.246
miR-144-5p				28.134	31.999	33.138
miR-145-3p				33.380	30.718	33.532

miR-145-5p	22.779	22.309	22.190	22.717	21.175	23.411
miR-146a-5p	30.917	32.999	27.377	22.768	19.153	26.670
miR-146b-5p	29.077	29.628	29.404	23.275	20.554	23.231
miR-148a-3p				28.255	29.459	32.710
miR-148b-5p				32.596	30.708	33.930
miR-149-5p				27.100	23.484	27.083
miR-150-5p	30.215	29.092	28.188	21.018	27.012	24.751
miR-151a-3p	31.853	29.978	28.287	25.254	23.229	25.754
miR-151a-5p	32.790	31.775	29.667	26.864	25.544	28.876
miR-152-3p	28.455	27.790	27.332	26.047	24.738	26.877
miR-155-5p	26.626	28.374	25.309	24.316	22.392	23.713
miR-15b-5p	28.990	30.484	28.468	26.112	26.553	30.017
miR-16-5p	26.281	28.392	26.619	22.474	22.776	24.305
miR-17-5p	26.854	27.814	26.397	22.960	23.749	25.344
miR-181a-2-3p				30.178	26.538	33.144
miR-184				31.046	28.873	29.943
miR-186-5p	29.143	29.135	28.449	23.538	21.575	24.443
miR-18a-3p				32.536	30.964	33.217
miR-18a-5p				29.189	31.903	30.928
miR-191-5p	24.714	25.389	23.734	20.986	19.540	19.908
miR-192-5p	33.030	32.979	33.061	28.105	32.294	30.482
miR-193a-3p	32.460	33.767	31.198	31.318	29.048	31.329
miR-193a-5p	28.850	28.934	25.893	27.384	24.127	29.348
miR-193b-3p	25.525	26.432	23.446	20.361	18.256	19.565
miR-193b-5p				28.935	27.509	31.269
miR-195-5p	30.169	30.849	29.980	27.393	26.720	30.457
miR-196b-5p	30.431	32.554	29.819	27.007	31.968	27.817
miR-197-3p	24.849	25.397	23.597			
miR-199a-3p	26.654	26.768	25.773			
miR-199-3p				23.048	23.140	26.986
miR-19a-3p	31.976	32.308	30.577	27.294	28.048	31.526
miR-19b-1-5p				32.270	30.202	33.022
miR-19b-3p	25.138	25.348	25.120	21.992	22.520	25.238
miR-203a-3p				30.949	30.209	32.308
miR-204-5p				27.074	25.803	31.128
miR-20a-5p	28.903	29.264	28.513	22.808	25.355	26.782
miR-20b-5p	30.437	30.817	29.898	27.764	28.532	30.979
miR-210-3p				23.558	21.146	26.966
miR-212-3p	31.372	30.857	30.163	30.326	25.742	26.168
miR-21-3p				30.970	27.675	32.171
miR-214-3p	24.629	24.818	24.138	22.959	21.412	23.207
miR-214-5p				30.055	28.318	29.880
miR-21-5p	24.865	24.900	24.102	23.106	21.221	24.968
miR-218-5p	28.271	30.127	27.974	25.278	22.498	25.835
miR-221-3p	27.133	26.934	24.761	25.529	24.146	26.777
miR-222-3p	22.731	23.027	20.190	18.156	18.239	21.066

miR-222-5p				24.473	27.147	29.027
miR-223-3p	31.194	31.307	29.296	20.599	26.209	25.938
miR-224-5p	32.878	31.482	30.403	27.381	26.193	28.950
miR-24-3p	22.440	23.044	20.994	19.743	17.489	19.853
miR-25-3p	30.173	32.644	31.147	27.079	26.914	30.319
miR-26a-1-3p				32.585	29.493	31.169
miR-26a-5p	29.971	30.402	29.098	24.331	23.546	28.627
miR-26b-5p	32.241	32.574	33.751	27.036	26.314	33.675
miR-27a-3p	28.956	30.570	28.273	27.430	26.159	28.082
miR-27a-5p				27.665	26.944	31.855
miR-27b-3p	29.152	30.137	28.095	29.152	26.451	30.844
miR-28-3p	28.619	29.068	27.664	24.634	23.683	26.976
miR-28-5p	29.648	30.745	30.045	27.989	26.378	30.022
miR-29a-3p	24.940	25.883	23.575	22.800	21.949	23.120
miR-29b-3p	32.351	32.141	30.109			
miR-301a-3p	31.332	30.558	30.790	27.255	27.371	32.097
miR-30a-3p	27.949	25.697	26.215	24.763	21.179	24.375
miR-30a-5p				25.712	24.180	27.510
miR-30b-5p	26.609	26.962	25.605	24.054	23.276	27.721
miR-30c-5p	26.884	27.393	25.483	23.387	22.803	27.533
miR-30e-3p	27.935	26.950	26.836	24.389	21.677	23.874
miR-31-3p				24.709	20.904	26.422
miR-31-5p	25.243	26.844	22.949	21.907	18.242	24.299
miR-320a	26.661	26.624	24.441	22.582	20.698	22.814
miR-320b				29.178	28.041	31.618
miR-323a-3p	29.755	29.528	28.539	29.616	27.281	29.514
miR-324-3p	32.959	33.469	31.012	27.290	23.258	28.984
miR-324-5p	31.205	31.040	31.818	30.350	28.951	33.124
miR-328-3p	29.582	29.966	27.360	27.256	27.136	32.231
miR-331-3p	27.422	28.754	25.140	23.461	22.215	27.526
miR-335-3p				28.683	25.440	31.973
miR-335-5p	30.792	31.205	30.758	28.335	26.281	30.770
miR-339-3p				29.019	26.077	26.948
miR-342-3p	25.239	24.767	24.314			
miR-345-5p	30.164	31.226	29.688	26.633	26.453	27.683
miR-34a-3p				27.029	25.532	28.031
miR-34a-5p	29.538	30.190	28.133	27.374	25.266	29.783
miR-34b-3p	32.389	32.314	30.124	26.948	27.009	28.596
miR-365a-3p	26.536	27.306	25.931			
miR-362-5p				30.079	28.258	32.719
miR-370-3p	30.287	30.704	27.042	26.893	23.582	26.290
miR-374a-5p	30.201	31.198	29.596	26.290	25.276	31.539
miR-374b-5p				24.630	25.707	29.947
miR-376a-3p	29.009	29.110	27.451	27.178	24.466	24.928
miR-376c-3p	29.352	29.401	28.389	25.592	23.379	26.193
miR-378				30.586	26.771	29.748

miR-379-5p				28.734	27.297	31.975
miR-382-5p				24.217	23.273	26.656
miR-409-3p	26.915	24.843	22.177	22.536	19.773	21.902
miR-410-3p				28.233	28.253	29.621
miR-411-5p	31.444	33.799	29.312	26.437	26.694	28.429
miR-424-3p				29.260	28.397	30.986
miR-424-5p				31.880	31.459	33.843
miR-425-3p				28.874	28.044	30.120
miR-432-5p	30.053	27.826	26.572	26.020	23.585	26.327
miR-433-3p	31.189	32.675	29.451	27.927	27.787	32.476
miR-451a				24.903	29.280	29.009
miR-452-5p				28.952	26.204	29.798
miR-454-3p	29.909	32.142	30.032	24.932	24.286	27.791
miR-455-5p				31.082	29.953	32.010
miR-483-5p	30.980	31.274	28.054			
miR-484	25.853	26.144	24.115			
miR-485-3p	31.566	32.755	28.810			
miR-487b-3p	33.961	32.973	32.390	28.339	26.946	32.036
miR-493-3p	33.640	33.568	31.981	30.044	30.347	29.712
miR-494-3p	30.186	30.635	29.264	26.960	28.281	29.373
miR-495-3p				27.842	27.423	30.585
miR-505-5p				30.222	30.910	33.306
miR-532-3p	30.251	29.982	28.892	28.301	25.376	28.842
miR-532-5p	30.237	30.405	29.268	27.015	24.510	28.372
miR-539-5p	29.946	29.728	28.413	26.182	24.448	29.109
miR-542-5p				31.446	28.813	31.014
miR-543				30.063	29.154	32.326
miR-548c-3p				28.447	33.044	30.173
miR-574-3p	25.066	24.762	23.343	21.977	20.413	21.764
miR-590-3p				31.361	27.966	32.715
miR-590-5p				29.874	27.778	30.780
miR-597-5p				31.988	32.990	31.655
miR-603				27.107	31.972	31.562
miR-625-3p	29.982	31.118	26.951	26.646	26.342	26.742
miR-628-3p				28.104	28.008	29.708
miR-628-5p				29.555	28.801	29.761
miR-629-3p				30.023	27.218	29.351
miR-652-3p				31.156	31.521	32.972
miR-655-3p				31.403	30.832	32.248
miR-660-5p				27.863	25.222	29.517
miR-664a-3p				24.898	23.298	27.964
miR-708-5p	31.953	32.549	29.167	29.424	25.487	28.763
miR-7-1-3p				27.282	25.564	27.531
miR-720	28.668	25.901	22.892	19.712	19.262	23.336
miR-744-5p	32.554	32.900	27.926	28.801	25.986	31.534
miR-766-3p				29.574	25.603	28.261

miR-886-3p				24.268	24.957	32.232
miR-886-5p	27.241	27.573	25.211	21.201	22.045	26.629
miR-889-3p				29.891	30.309	32.851
miR-92a-3p	27.020	28.069	25.992	24.978	24.937	26.833
miR-93-3p	31.401	29.523	28.037	28.065	25.105	27.447
miR-93-5p				26.327	26.382	30.763
miR-99a-5p	24.567	24.382	22.397			
miR-99b-3p	31.995	30.758	28.495	28.848	25.274	25.362

Table S2: Target Pathways of miRNAs Carried Only by ADSC-EVs.

The list of miRNAs only present in ADSC-EVs was analyzed by mirPath v.3 [33]. The columns show the p-value, the number of target genes in the pathway, and the number of miRNAs involved. Highlighted pathways have been selected and showed in Figure 3.

KEGG pathway	p-value	genes	miRNAs
TGF-beta signaling pathway	1.26E-05	64	48
ErbB signaling pathway	4.64E-05	73	52
PI3K-Akt signaling pathway	0.000147	244	61
ECM-receptor interaction	0.000327	60	47
Adherens junction	0.000953	60	46
Regulation of actin cytoskeleton	0.001317	156	59
MAPK signaling pathway	0.005229	177	57
Wnt signaling pathway	0.006119	101	56
HIF-1 signaling pathway	0.028221	76	50
Pathways in cancer	0.000766	283	62
Ras signaling pathway	4.03E-05	166	57
Focal adhesion	3.21E-05	158	56
Proteoglycans in cancer	1.59E-10	156	61
Rap1 signaling pathway	0.001452	153	52
Endocytosis	0.001868	147	59
cAMP signaling pathway	0.038672	137	55
cGMP-PKG signaling pathway	0.014654	117	57
Hippo signaling pathway	3.31E-06	116	54
Oxytocin signaling pathway	0.005343	114	55
Signaling pathways regulating pluripotency of stem cells	8.41E-05	107	56
Adrenergic signaling in cardiomyocytes	0.00331	105	54
FoxO signaling pathway	8.41E-05	103	56
Ubiquitin mediated proteolysis	0.002169	101	52
Axon guidance	3.31E-06	99	52
Tight junction	0.048392	98	52
Platelet activation	0.001511	95	50
Dopaminergic synapse	0.009942	94	57
AMPK signaling pathway	0.033535	89	59
Glutamatergic synapse	4.87E-05	88	51
Thyroid hormone signaling pathway	0.024724	84	52

Sphingolipid signaling pathway	0.018238	83	48
Cholinergic synapse	0.014087	81	53
Choline metabolism in cancer	0.000395	79	54
T cell receptor signaling pathway	0.033535	76	48
Estrogen signaling pathway	0.000375	75	52
Retrograde endocannabinoid signaling	0.044714	72	52
Morphine addiction	0.005343	67	50
Fc gamma R-mediated phagocytosis	0.022426	67	46
Gap junction	0.001736	66	48
GnRH signaling pathway	0.044714	66	51
Dilated cardiomyopathy	0.024724	65	47
Prostate cancer	0.033535	64	53
GABAergic synapse	0.014654	63	49
Bacterial invasion of epithelial cells	0.000987	60	43
Phosphatidylinositol signaling system	0.044714	58	51
Melanoma	0.005517	56	49
Chronic myeloid leukemia	0.017764	56	51
Gastric acid secretion	0.028221	56	46
Renal cell carcinoma	0.001317	55	47
Prolactin signaling pathway	0.000541	54	46
B cell receptor signaling pathway	0.028221	54	47
Arrhythmogenic right ventricular cardiomyopathy (ARVC)	0.018973	53	47
Fc epsilon RI signaling pathway	0.038672	53	44
Glioma	4.87E-05	52	50
Pancreatic cancer	0.003026	52	46
Long-term depression	3.31E-06	51	45
p53 signaling pathway	0.033535	51	46
Thyroid hormone synthesis	0.024724	50	48
Long-term potentiation	0.038672	50	49
mTOR signaling pathway	0.008471	48	49
Amphetamine addiction	0.008471	47	49
Colorectal cancer	0.024379	46	42
Acute myeloid leukemia	0.014654	43	45
Non-small cell lung cancer	0.038672	41	49
N-Glycan biosynthesis	0.003026	36	39
Cocaine addiction	0.00284	34	48
Nucleotide excision repair	0.044714	34	32
Circadian rhythm	0.006206	27	43
Mucin type O-Glycan biosynthesis	3.92E-15	26	33
Dorso-ventral axis formation	0.024379	23	41
Prion diseases	0.024379	18	29
Glycosaminoglycan biosynthesis - heparan sulfate / heparin	0.024379	16	19
Fatty acid biosynthesis	3.20E-05	10	20

Table S3: Target Pathways of miRNAs Carried by ADSC-EVs and BMSC-EVs.

The list of miRNAs present in ADSC-EV and BMSC-EV was analyzed by mirPath v.3 [33]. The columns show the p-value, the number of target genes in the pathway, and the number of miRNAs involved. Highlighted pathways have been selected and showed in Figure 3.

**KEGG pathway**	**p-value**	**genes**	**miRNAs**
ECM-receptor interaction	1.12E-11	63	72
TGF-beta signaling pathway	1.38E-06	65	78
Wnt signaling pathway	2.87E-05	112	87
PI3K-Akt signaling pathway	0.000117	247	89
ErbB signaling pathway	0.001196	70	86
Mucin type O-Glycan biosynthesis	2.32E-13	26	50
Proteoglycans in cancer	8.80E-13	160	88
Pathways in cancer	1.12E-11	305	91
Hippo signaling pathway	1.18E-08	122	86
Rap1 signaling pathway	2.75E-07	167	92
Signaling pathways regulating pluripotency of stem cells	1.08E-06	112	90
Ras signaling pathway	3.18E-06	169	92
Axon guidance	3.18E-06	102	86
Lysine degradation	1.13E-05	40	74
Glioma	1.25E-05	53	80
Fatty acid biosynthesis	2.57E-05	10	35
N-Glycan biosynthesis	4.88E-05	37	60
MAPK signaling pathway	7.43E-05	192	92
Glycosphingolipid biosynthesis - ganglio series	8.44E-05	12	36
Renal cell carcinoma	0.000117	55	80
Long-term depression	0.000118	48	79
Morphine addiction	0.000146	67	83
Focal adhesion	0.000218	155	90
Adherens junction	0.000254	60	82
Circadian entrainment	0.000262	75	83
Endocytosis	0.000283	152	86
Bacterial invasion of epithelial cells	0.000513	60	81
Protein processing in endoplasmic reticulum	0.000549	121	82
AMPK signaling pathway	0.000833	96	83
Glutamatergic synapse	0.000833	85	83
Neurotrophin signaling pathway	0.001058	94	92
Adrenergic signaling in cardiomyocytes	0.001058	106	86
Inflammatory mediator regulation of TRP channels	0.001058	77	80
Arrhythmogenic right ventricular cardiomyopathy (ARVC)	0.001114	57	73
Melanoma	0.00129	58	72
Small cell lung cancer	0.001337	68	75
Pancreatic cancer	0.001546	52	77
Regulation of actin cytoskeleton	0.001645	155	86
Gap junction	0.001877	66	81
Thyroid hormone signaling pathway	0.001888	89	85
Cholinergic synapse	0.002104	84	84

FoxO signaling pathway	0.002104	100	80
Endometrial cancer	0.00301	42	79
Acute myeloid leukemia	0.00301	46	77
Choline metabolism in cancer	0.003079	78	84
Colorectal cancer	0.003079	50	79
Glycosaminoglycan biosynthesis - keratan sulfate	0.003271	13	26
mTOR signaling pathway	0.003632	49	79
Fc gamma R-mediated phagocytosis	0.003632	70	78
Phosphatidylinositol signaling system	0.005522	61	81
Oxytocin signaling pathway	0.006257	116	87
Non-small cell lung cancer	0.006257	43	77
Chronic myeloid leukemia	0.01023	55	78
Glycosaminoglycan biosynthesis - heparan sulfate / heparin	0.012592	19	39
cAMP signaling pathway	0.013119	141	91
Melanogenesis	0.013119	75	84
Basal cell carcinoma	0.013119	44	71
Sphingolipid signaling pathway	0.013489	85	83
Prostate cancer	0.013489	66	79
Prolactin signaling pathway	0.014217	52	83
Central carbon metabolism in cancer	0.017781	51	77
Retrograde endocannabinoid signaling	0.018097	73	83
Estrogen signaling pathway	0.02011	71	86
cGMP-PKG signaling pathway	0.02676	117	91
Fatty acid metabolism	0.030477	33	57
Hedgehog signaling pathway	0.032833	40	61
GABAergic synapse	0.038066	62	80
mRNA surveillance pathway	0.042254	66	78
Amoebiasis	0.046394	73	73
Shigellosis	0.047824	45	73
Prion diseases	0.047824	17	45
Platelet activation	0.049165	89	86
Alanine aspartate and glutamate metabolism	0.02475	28	58

Table S4: Target Pathways of miRNAs Carried Only by BMSC-EVs.

The list of miRNAs only present in BMSC-EV was analyzed by mirPath v.3 [33]. The columns show the p-value, the number of target genes in the pathway, and the number of miRNAs involved. Highlighted pathways have been selected and showed in Figure 3.

KEGG pathway	p-value	genes	miRNAs
ECM-receptor interaction	1.19E-45	36	10
PI3K-Akt signaling pathway	2.83E-06	115	14
ErbB signaling pathway	0.000232	36	13
Adherens junction	0.000546	29	10
MAPK signaling pathway	0.006873	80	13
Wnt signaling pathway	0.015306	42	13

Glioma	1.47E-09	32	13
Prion diseases	9.34E-08	3	4
Proteoglycans in cancer	1.01E-07	72	13
Amoebiasis	2.83E-06	39	12
Focal adhesion	3.97E-05	76	14
Melanoma	0.000135	32	13
mTOR signaling pathway	0.000359	28	12
Ras signaling pathway	0.000462	75	14
Pathways in cancer	0.003595	115	13
Rap1 signaling pathway	0.003595	68	14
Phosphatidylinositol signaling system	0.003698	26	9
Lysine degradation	0.003698	15	11
Renal cell carcinoma	0.003698	27	13
Platelet activation	0.004725	42	13
FoxO signaling pathway	0.005798	47	12
Non-small cell lung cancer	0.006873	23	11
Long-term potentiation	0.006873	27	13
Estrogen signaling pathway	0.008066	31	11
Axon guidance	0.010747	37	12
Pancreatic cancer	0.011161	22	11
Protein digestion and absorption	0.011161	33	12
Glycosaminoglycan biosynthesis - heparan sulfate / heparin	0.013868	10	7
Choline metabolism in cancer	0.013868	37	13
Signaling pathways regulating pluripotency of stem cells	0.013868	44	14
Prostate cancer	0.014845	33	14
Colorectal cancer	0.021417	24	10
Mucin type O-Glycan biosynthesis	0.024404	10	7
Transcriptional misregulation in cancer	0.025868	52	12
Thyroid hormone signaling pathway	0.031291	37	12
Small cell lung cancer	0.038298	30	11
Regulation of actin cytoskeleton	0.041897	65	13
